# Megabase-scale methylation phasing using nanopore long reads and NanoMethPhase

**DOI:** 10.1186/s13059-021-02283-5

**Published:** 2021-02-22

**Authors:** Vahid Akbari, Jean-Michel Garant, Kieran O’Neill, Pawan Pandoh, Richard Moore, Marco A. Marra, Martin Hirst, Steven J. M. Jones

**Affiliations:** 1 Canada’s Michael Smith Genome Sciences Centre, BC Cancer, Vancouver, British Columbia Canada; 2 Department of Medical Genetics, University of British Columbia, Vancouver, British Columbia Canada; 3 Department of Microbiology and Immunology, Michael Smith Laboratories, University of British Columbia, Vancouver, British Columbia Canada

**Keywords:** Nanopore sequencing, Allele-specific methylation, Phasing, NanoMethPhase

## Abstract

The ability of nanopore sequencing to simultaneously detect modified nucleotides while producing long reads makes it ideal for detecting and phasing allele-specific methylation. However, there is currently no complete software for detecting SNPs, phasing haplotypes, and mapping methylation to these from nanopore sequence data. Here, we present NanoMethPhase, a software tool to phase 5-methylcytosine from nanopore sequencing. We also present SNVoter, which can post-process nanopore SNV calls to improve accuracy in low coverage regions. Together, these tools can accurately detect allele-specific methylation genome-wide using nanopore sequence data with low coverage of about ten-fold redundancy.

**Supplementary Information:**

The online version contains supplementary material available at 10.1186/s13059-021-02283-5.

## Introduction

Somatic cells of diploid organisms comprise two alleles for each gene, and most genes are expressed from both alleles [1]. However, some genes only express from one allele, often in a lineage- or tissue-specific manner [1]. Various mechanisms can control mono-allelic expression (MAE), including DNA polymorphisms at regulatory regions, and differential epigenetic modifications [1, 2]. Imprinting is a specific type of MAE in which the expressed allele is defined based upon the parent of origin through epigenetic modifications, primarily DNA methylation at CpG sites located in imprinting control regions (ICRs) [2]. In addition to imprinting, MAE can be a result of random mono-allelic expression (RME) where allelic choice randomly occurs somatically in a tissue- and cell type-specific and non-parent of origin manner, and X chromosome inactivation (XCI) is a well-established RME [3]. Both RME and imprinting have substantial roles in normal growth and development, behavior, and metabolism [3, 4]. Aberrant DNA methylation at ICRs results in various developmental disorders and loss of imprinting is frequently observed in human tumors [2, 5, 6].

Detection of allele-specific methylation (ASM) requires profiling both allele-specific SNPs and DNA methylation on the same or linked data. The current gold standard approach to study DNA methylation is whole-genome bisulfite sequencing (WGBS) [7, 8]. Discrimination of methylated and unmethylated CpGs in WGBS is based on bisulfite conversion. During bisulfite treatment, unmethylated cytosine converted to uracil which then replaced by thymidine through downstream PCR reactions prior to short-read sequencing. Subsequent short-read sequencing and mapping of reads to the reference genome can detect DNA methylation genome-wide [7, 8]. However, short-read pairs typically cannot span adjacent allele-specific SNPs in regions of low variant density. Moreover, bisulfite conversion is a challenging molecular protocol, which introduces errors both from the harsh chemical treatment and the difficulty of mapping converted reads to the reference genome [7, 9]. Third-generation long-read sequencing provided by Oxford Nanopore Technologies (ONT) and Pacific Biosciences single-molecule real-time sequencing not only sequences DNA but can also detect DNA methylation through picoampere signal intensities and polymerase kinetics, respectively [10, 11]. Long reads produced by these technologies can span over several kilobases and can resolve the phasing problem at regions of low SNP density. However, the higher sequencing error rate can impede the accurate detection of SNPs and the haplotype phasing of long reads. Additionally, Pacific Biosciences is prohibitively expensive as it requires very high coverage (250× per strand) to confidently detect 5-methylcytosine which makes it impractical on a mammalian size genome [12].

In nanopore sequencing, as nucleic acids are propelled through a protein nanopore embedded in an electrically resistant membrane, the chemical composition of the approximately five nucleotides (5-mers) present in the narrowest region of the pore defines the measurable current signal across the membrane (pore version R9.4) [13]. These distinctive signal characteristics are interpretable by a trained artificial neural network during typical base calling. A similar approach is used to call 5-methylcytosine at CpG sites by tools such as Megalodon [14], Nanopolish [10], DeepSignal [15], DeepMod [16], and SignalAlign [17]. Therefore, in nanopore sequencing, both DNA sequences and modifications are detectable using raw signal information and there is no need for bisulfate conversion and PCR amplification prior to sequencing [16, 18]*.* Statistically based approaches are also available to detect base modifications using comparative tests on signals from pairwise samples (e.g., wild-type and knock out), Nanoraw [19] and NanoMod [20]. Statistically based approaches typically call all detectable base modifications between the samples without distinction of the specific type of modification [20]. Model approaches can potentially differentiate several types of modification assuming the model used was trained accordingly. These sets of tools can be used together on the same signal data to generate both DNA sequence and CpG methylation, which is ideal for detecting ASM. Using these features, Gigante et al. [21] successfully used known SNPs from parental mice to phase methylation in the F1 and detect ASM. However, there is a lack of a straightforward workflow and software tools to phase both reads and methylation data from nanopore sequencing. More importantly, an approach which can phase nanopore reads using only nanopore sequencing, without having to detect SNVs using other platforms or known parental SNVs, has not been previously shown.

Here, we have developed a workflow and associated software, SNVoter and NanoMethPhase, to detect ASM from a single sample using only nanopore sequence data with redundant sequence coverage as low as about 10×. We called SNVs from nanopore sequencing data using Clair [22]. Clair is designed to call germline small variants from nanopore reads based on pileup format, and the authors demonstrated its superiority over other pileup-based tools [22]. We demonstrated that we can improve SNV detection significantly by using SNVoter. Subsequently, we phased the SNVs detected from nanopore reads using WhatsHap [23] v 0.18 and used our tool, NanoMethPhase, to phase both the sequence reads and the CpG methylation. Using a normal human B-lymphocyte cell line (NA19240), we demonstrated that NanoMethPhase can accurately detect ASM and parent of origin when trio data (i.e., using known SNPs from mother, father, and child) is available. Moreover, NanoMethPhase detected ASM for this sample using only nanopore sequence, showing high concordance with the trio-based phasing. We further demonstrated the detection of ASM with about 10× coverage using a Colo829BL B-lymphoblast cell line.

## Results

### Benchmarking of nanopore methylation calling software

**Fig. 1:**
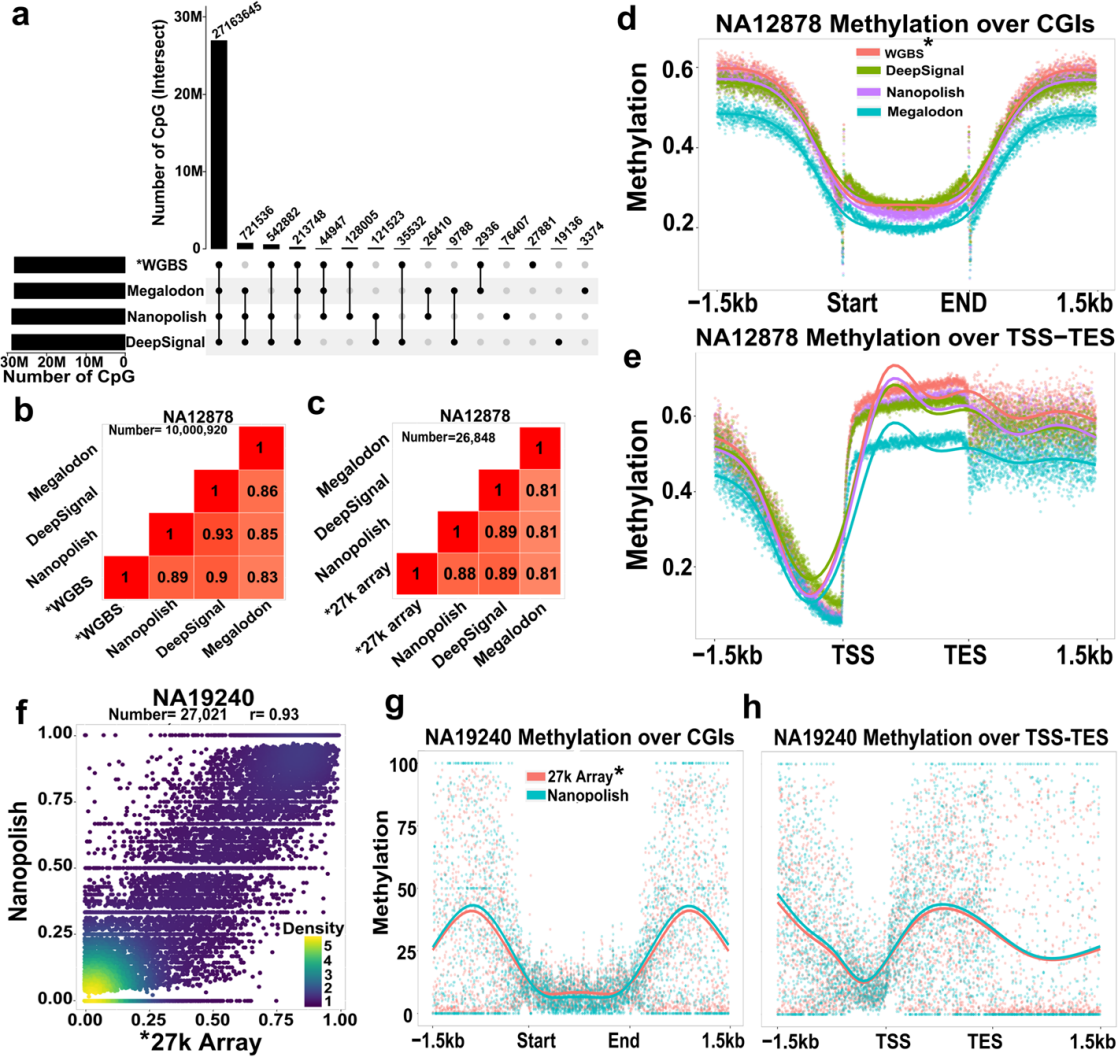
Methylation calling from nanopore data and comparison to gold standard platforms. **a** UpSet plot of the intersections of CpG sites detected using DeepSignal, Nanopolish, Megalodon, and whole-genome bisulfite sequencing (WGBS) for NA12878. Because Encode WGBS workflow used index generated from GRCh38 assembly with alternative contiguous removed, only CpG methylations on main chromosomes (1–22 and X) were considered. **b**, **c** Pearson correlation matrix of methylation levels from the tools with WGBS (**b**) and Illumina’s 27k methylation array (**c**). For comparison to WGBS, only CpGs with at least 5 calls were considered. The number represents common CpGs in all methods. **d** Distribution of methylation over CpG islands (CGIs). **e** Distribution of methylation at transcription start (TSS) and end sites (TES). **f** Scatter plot of methylation level obtained from Nanopolish and Illumina’s 27k methylation array for NA19240 sample. Pearson correlation coefficient presented as *r*. **g**–**h** Distribution of methylation over common CpGs between Nanopolish and 27k array at CpG islands (CGIs) (**g**) or transcription start (TSS) and end sites (TES) (**h**). Gold standard methods for CpG methylation detection are indicated by an asterisk

We benchmarked three tools able to detect CpG methylation from nanopore sequencing using a pre-trained model: Nanopolish [10], Megalodon [14], and DeepSignal [15]. The CpG methylation calls obtained were compared to matching data from WGBS and Illumina Infinium HumanMethylation 27 BeadChip (henceforth, 27k methylation array). We used 12 MinION flow cells (~ 12× coverage) of publicly available nanopore sequencing data for NA12878 [24] (Additional file 1) to compare with ENCODE WGBS data (ENCFF835NTC) and 27k methylation array [25] (GSM670984) data for this cell line. DeepSignal, Nanopolish, and Megalodon were able to call methylation for ~ 30M, ~ 29.7M, and ~ 29.2M CpG sites, respectively. Because ENCODE WGBS workflow used an index generated from the GRCh38 assembly with alternative contigs removed, only called CpG sites on the main chromosomes (1–22 and X) were considered. DeepSignal, Nanopolish, Megalodon, and WGBS were able to call methylation for 28.827M, 28.825M, 28.186M, and 28.159M CpG sites on the main chromosomes, respectively (Fig. 1a). Nanopolish and DeepSignal showed a higher correlation with WGBS (Fig. 1b, 0.89 for Nanopolish and 0.90 for DeepSignal; Additional file 2: Fig. S1a-f) and 27k methylation array (Fig. 1c, 0.88 for Nanopolish and 0.89 for DeepSignal) compared to Megalodon (0.83 with WGBS and 0.81 with 27k array). The correlation with WGBS was also evaluated based on CpG density and at each genomic region (Additional file 2: Fig. S1g, h). These analyses showed that the correlation is lower at low- and high-density CpG sites (< 4% CpG per 2 kb and > 8% CpG per 2 kb) and DeepSignal works better at low dense CpG sites while Nanopolish gives better correlation at high-density CpG regions. Moreover, the best correlation with WGBS is obtained at enhancers and promoters, while repeats and intergenic regions had the lowest correlation. In agreement with correlation analysis based on CpG density, Nanopolish showed slightly better correlation at CpG islands while DeepSignal worked better in other regions. The distribution of methylation levels obtained from Nanopolish and DeepSignal closely followed the trend of WGBS values across CpG islands (CGIs, Fig. 1d). They also outperformed Megalodon around transcription start (TSS) and end sites (TES) (Fig. 1e), with Nanopolish showing closer concordance with WGBS near TSS. Consequently, we selected Nanopolish as the most appropriate tool to call methylation, with the added benefits that were faster processing time and also fewer pre-processing steps.

To further validate methylation calling using Nanopolish, we compared methylation calling for NA19240 (ERR3046935 or run 1; Additional file 1) [26] with the 27k methylation array (GSM671076) [25]. They showed 0.93 Pearson correlation between methylation frequencies from Nanopolish and beta-values from 27k methylation array data (Fig. 1f). The distribution of methylation calls showed the same close concordance across CGIs (Fig. 1g) and TSS-TES (Fig. 1h).

As shown in Fig. 1a, Nanopolish and DeepSignal called methylation at considerably more CpG sites compare to WGBS. Nanopolish and DeepSignal had ~ 945,000 and ~ 871,000 CpGs not present in WGBS, respectively, and ~ 843,000 of them were common to both Nanopolish and DeepSignal. Approximately 50% of these nanopore-specific CpGs mapped to satellite repeats (Additional file 2: Fig. S2a) which are complex repeats mostly found in centric and pericentric regions. Of satellite repeat types, approximately 98% of the nanopore-specific CpGs mapped to ALR/Alpha repeats (Additional file 2: Fig. S2b). This demonstrates the advantages of nanopore sequencing in mapping to complex repeat regions.

### High false positives in variant calling results

SNPs are the most commonly occurring variants and are routinely used to phase reads between haplotypes. Thus, to phase nanopore reads and CpG methylation, detection of SNPs is first required. In order to evaluate single-sample variant calling from nanopore data, we used Clair [22] to call SNVs on 20 flow cells of the NA12878 sample (24×; Additional file 1) described above and compared these calls to gold standard SNVs from the Genome in a Bottle database (GIAB, V3-3-2) [27]. Clair detected 6.712M SNVs, 3.711M of which exceeded the variant call quality filtering threshold of 730 (Additional file 2: Fig. S3a). We then counted false positives (943,304), false negatives (223,581), true positives (2,768,084), and true negatives (2,777,463) to calculate accuracy, precision, recall, and F1 score, measured at 82.6%, 74.6%, 92.5%, and 82.6%, respectively. The majority (90%) of true SNVs were determined in Clair as high-quality SNV calls. However, there were still a considerable number of false positives which can confound downstream analysis such as phasing. Therefore, we sought to improve the accuracy and the precision and reduce the false-positive rate as much as possible while retaining true positives.

### Base qualities and mutation frequencies are informative

**Fig. 2:**
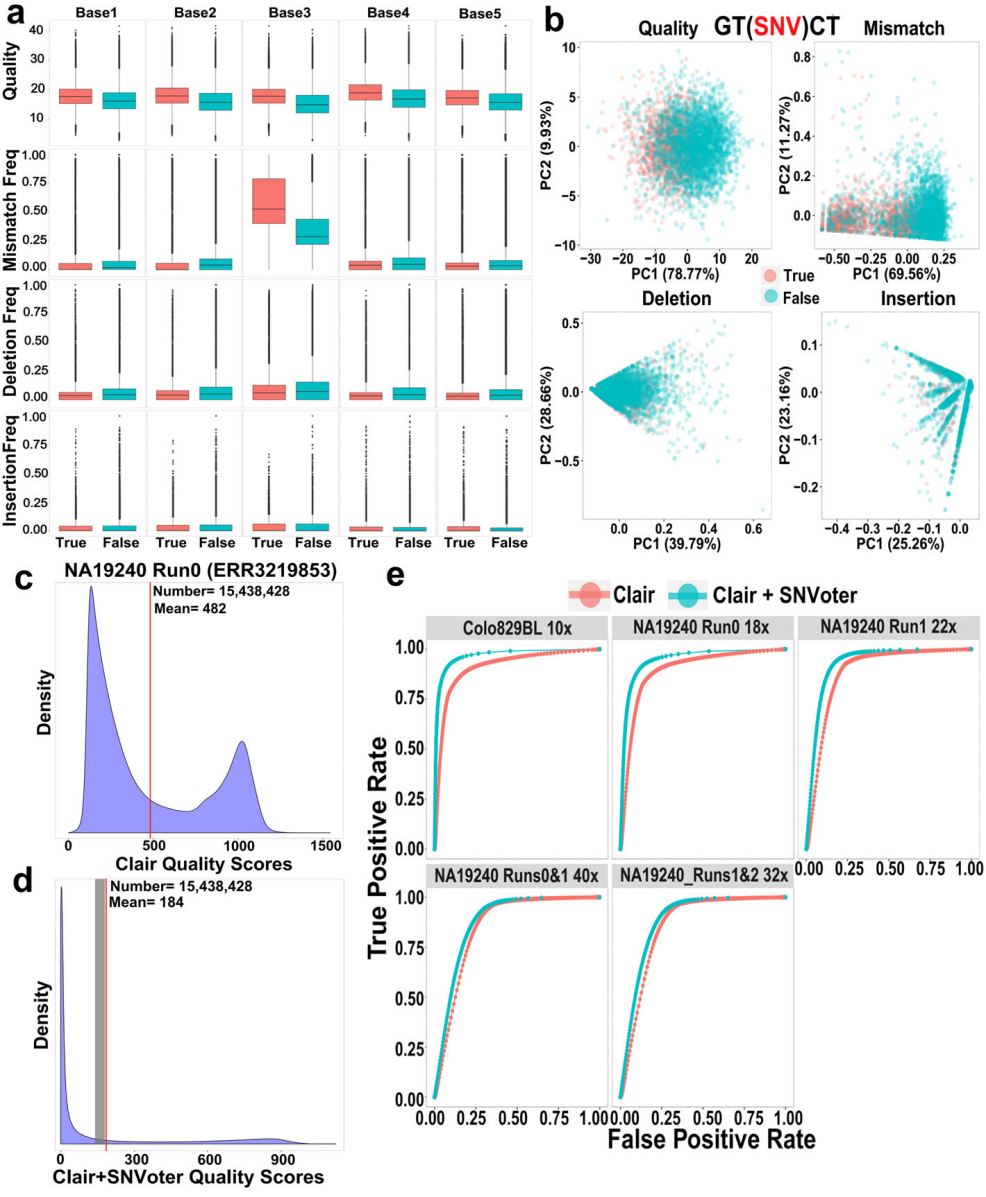
Improvement in Clair SNV calls from nanopore data. **a** Base quality and mutation frequencies obtained for 1 million randomly selected 5-mers with SNV in the base 3 position (1:1 true positives to false positives). **b** PCA analysis of the reference 5-mer GTACT shows separation of true positive from false positives based on quality scores and mismatch frequencies. **c** Clair variant calling quality distribution for the NA19240 run 0 sample. **d** Quality distribution upon normalization of Clair’s qualities using the weights given by SNVoter to each SNV. The highlighted region represents the optimal threshold area to filter out low-quality calls. **e** Receiver operating characteristic curves for SNV calling using Clair or using Clair+SNVoter for different coverage depths. NA19240 run 1, NA19240 run 0, and Colo829BL are processed by SNVoter using the model trained on NA12878 20FCs (24×). NA19240 runs 0&1 and NA19240 runs 1&2 is processed using the model trained on NA12878 whole dataset (44×)

Studies have demonstrated that in nanopore sequencing the signal distribution is driven mostly by the five to six bases that occupy the narrowest part of the pore [28]. During the base-calling process, base callers segment signals and translate them to the appropriate 5-mer using a pre-trained model. We hypothesized that if an SNV is a false call, the base caller must have been mistaken in calling the 5-mer, and therefore, additional sequencing errors should be found in the 5-mer windows adjacent to that SNV. Moreover, the Phred quality of called bases should be affected. To test this, we extracted Phred scores, mismatch, deletion, and insertion frequencies for SNVs where the Clair output agreed with the GIAB standard (true positives), and compared these with the false-positive SNVs. Indeed, higher mutation frequencies and lower base quality scores were observed in the adjacent 5-mer windows of false SNVs compared to true SNVs (Fig. 2a). Moreover, in a PCA analysis, they separated into two distinct groups where mismatch frequencies and base qualities seemed to have the highest effect on discriminating true-positive SNVs from false positives (Fig. 2b). These results demonstrate that base qualities and mutation errors in 5-mer windows are informative and can be used to further discriminate true positives and false positives in order to improve SNV calling from nanopore data.

### A neural network SNV classifier improves Clair SNV calls

We then used base quality and mutation frequencies to train an artificial neural network to discriminate true-positive SNVs from false calls (see the “Materials and methods” section), which we packaged into a tool we called SNVoter. Three different models were trained using various fold coverages from three datasets including NA12878 [24] (20 flow cells, 24×), NA12878 [24] (all flow cells, 44×), and HG003 [29] (80×). See additional file 1 for a full description of datasets used in our study. SNVoter classifies each 5-mer that includes an SNV. Since each base represents itself in five 5-mers (e.g., ATCG**A**CGTC will be represented in ATCG**A,** TCG**A**C, CG**A**CG, G**A**CGT, and **A**CGTC), the final prediction for each SNV is an average of all five predictions. Finally, we used the predictions from our classifier as weights, and for each SNV, we multiplied the variant call quality from Clair with its weight from our model to produce normalized quality scores.

To test the classifier and to obtain optimal quality threshold for normalized base quality scores, we used the NA19240 run 0 (whole dataset includes five PromethION runs, Additional file 1) basecalled nanopore sequencing dataset [26] (ERR3219853, 18× coverage) and variant calling data from the 1000 Genomes Project (1KGP) phase3 [30]. As presented in Fig. 2c and d, there is an obvious shift toward the low-quality region of the quality distribution plot after using SNVoter. This is largely due to the false-positive SNVs with high variant call quality from Clair that were assigned low weights by SNVoter. We plotted receiver operating characteristic curves (ROC) across a range of thresholds for normalized quality and used these to obtain the new optimal threshold (Fig. 2e). The ROC curve analysis demonstrates an improvement in discriminating true-positive SNV calls from false positives, and we determined the new optimal threshold to be the end of the first peak and the start of the valley (highlighted region in Fig. 2d. This threshold was further confirmed by more datasets we used that explained below). Other important metrics including accuracy, precision, recall, and F1 score are presented in Table 1 (for a complete table of different thresholds, see Additional file 3).

**Table 1:** Different metrics for SNV calls using Clair and Clair + SNVoter

Tool	Sample	QT	All TP	TP	FP	Acc	Pre	Rec	F1
C	Colo829BL	725	3,638,416	2,664,923	774,123	0.88	0.77	0.79	0.78
C+S	Colo829BL	160	3,638,416	2,851,683	336,594	0.93	0.89	0.84	0.87
C	NA19240 Run0	755	3,973,249	2,997,917	1,462,313	0.86	0.67	0.81	0.74
C+S	NA19240 Run0	170	3,973,249	3,160,297	956,846	0.90	0.77	0.86	0.81
C	NA19240 Run1	780	3,973,249	3,465,350	990,793	0.84	0.78	0.92	0.84
C+S	NA19240 Run1	350	3,973,249	3,436,275	658,067	0.88	0.84	0.91	0.87
C	NA19240 runs 1&2	800	3,973,249	3,644,703	812,335	0.84	0.82	0.96	0.88
C+S	NA19240 runs 1&2	180	3,973,249	3,595,674	683,727	0.86	0.84	0.94	0.89
C	NA19240 runs 0&1	820	3,973,249	3,639,922	750,137	0.85	0.83	0.95	0.89
C+S	NA19240 runs 0&1	170	3,973,249	3,592,467	653,594	0.85	0.85	0.94	0.89

*C* Clair, *S* SNVoter, *QT* quality threshold, *TP* true positive, *FP* false positive, *Acc* accuracy, *Pre* precision, *Rec* recall. All TP refers to all high-quality SNVs from 1KGP for NA19240 and from Strelka for Colo829BL Illumina sequencing

Finally, to comprehensively test SNVoter on different coverages, we used a Colo829BL sample which we sequenced (~10×), NA19240 run 1 [26] (ERR3219854 or ERR3046935. ~22×), NA19240 runs 1&2 (ERR3219854-5. ~32×), NA19240 runs 0&1 (ERR3219853-4. ~40×), and NA19240 4 runs (ERR3219854-7. ~65×). As presented in Fig. 2e and in Table 1, SNVoter can significantly improve SNV calls in data with up to 30× coverage (Clair variant call quality and normalized quality distributions are shown in Additional file 2: Fig. S3c). We used different models and random down-sampling of coverage for NA19240 4 runs (ERR3219854-7. ~ 65×), which did not provide any improvement when using SNVoter (Additional file 2: Fig. S3b). Overall, using SNVoter, we could raise the accuracy, precision, recall, and F1 using low coverage data to be comparable to that from high coverage data.

### Phasing of SNVs detected by nanopore sequencing

**Fig. 3:**
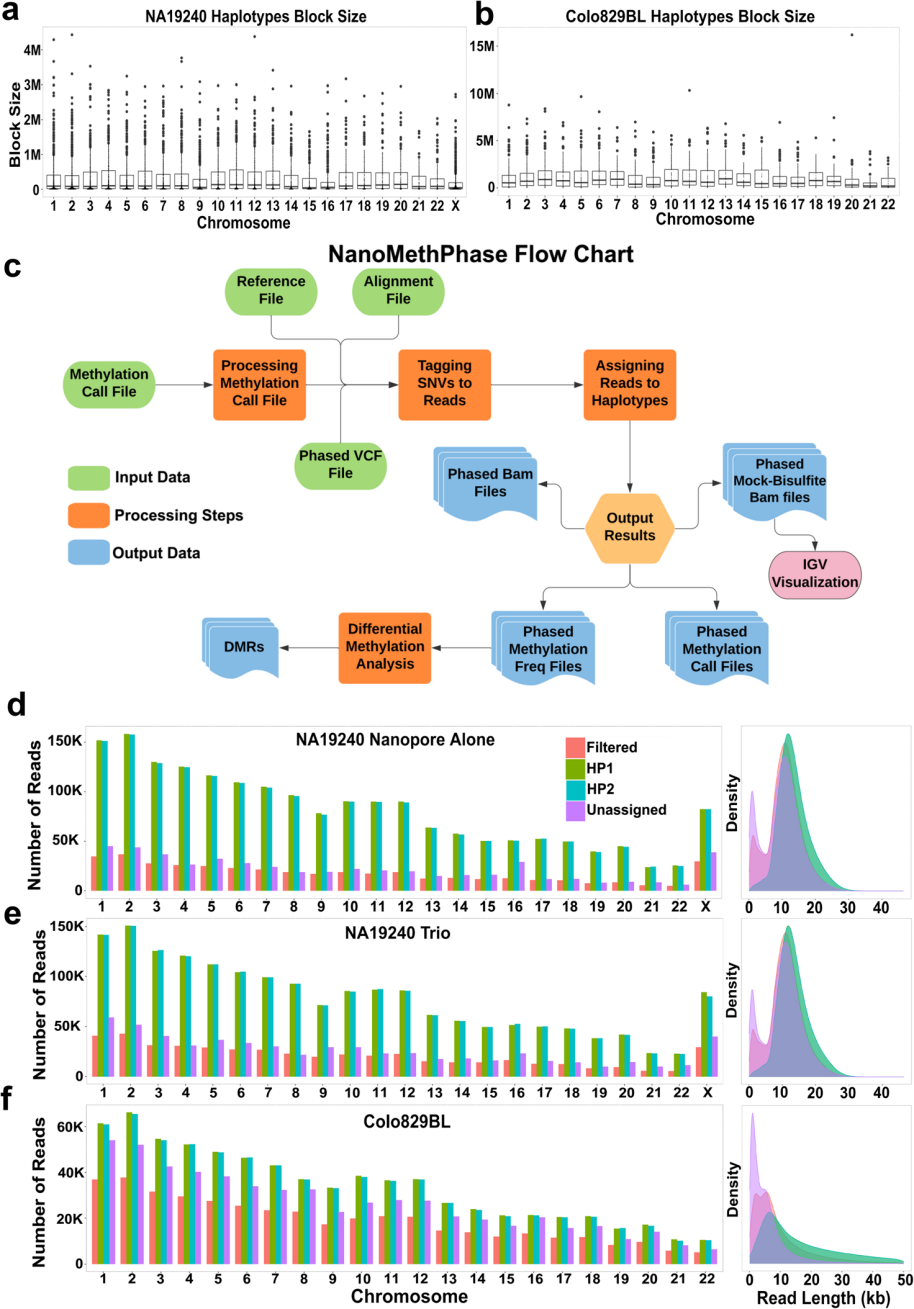
NanoMethPhase workflow and read phasing. **a**, **b** Haplotype block sizes following phasing of NA19240 and Colo829BL detected high-quality SNVs using WhatsHap. **c** NanoMethPhase workflow representing inputs, processing steps, and outputs. The output options can be requested independently to fit the needs. **d**–**f** Number of reads that were phased, filtered out, or could not be assigned to any phased SNV (left panel) and their length distribution (right panel, for ease in visualization reads with length < 50 kb are shown). **d** Obtained from NA19240 run 1 using nanopore phasing alone, **e** NA19240 run 1 trio phasing, and **f** Colo829BL sample. *NanoMethPhase phasing step ignores duplicated, QC failed, unmapped, and secondary reads. Supplementary reads also excluded by default but can be included as an optional parameter. The plots represent reads using default parameters

After normalizing Clair qualities using SNVoter and filtering out low-quality calls based on an optimized threshold (Table 1; Additional file 2: Fig. S3c) for NA19240 run 1 and Colo829BL samples, these SNVs were leveraged to phase nanopore reads. We phased SNVs using WhatsHap [31]. In the Colo829BL sample, ~ 37.5% of haplotype blocks were > 1 Mb (mean = 1116.5Kb, median = 588.3Kb), and in the NA19240 sample, ~ 9.25% of haplotype blocks were > 1 Mb (mean = 315.9Kb, median = 82.2Kb) (Fig. 3a, b). While the median read lengths were 8Kb and 12Kb for Colo829BL and NA19240 run 1, respectively, Colo829BL had numerous large reads. For example, 23% (678,900) of Colo829BL reads were > 20Kb while in NA19240 8.8% (513,373) of reads were > 20Kb, which resulted in a higher average read length in Colo829BL compared to NA19240 (Additional file 2: Fig. S4a and S4b). This can explain the numerous larger haplotype blocks in Colo829BL and highlights the value of longer reads, specifically in the proportion of all reads, for having a larger haplotype length.

### NanoMethPhase detects allele-specific methylation

The phased SNVs, alignment file, methylation call file from Nanopolish, and the reference genome are supplied to our tool, NanoMethPhase, to phase reads and CpG methylations (Fig. 3c; the “Materials and methods” section). In NA19240 run 1, out of 4.165 M reads which tagged with at least one phased SNV, 1.883M were assigned to the first haplotype (reference haplotype, HP1) and 1.871M were assigned to the second haplotype (alternative haplotype, HP2) (Fig. 3d). In the Colo829BL sample, out of 1.9 M reads which tagged with at least one phased SNV, 0.743M reads were assigned to HP1 and 0.738M reads were assigned to HP2 (Fig. 3f). In terms of speed, using 48 CPU cores, it took 10 h for NanoMethPhase to process the NA19240 run 1 sample (22×) and 5 h for the Colo829BL sample (10×).

**Fig. 4:**
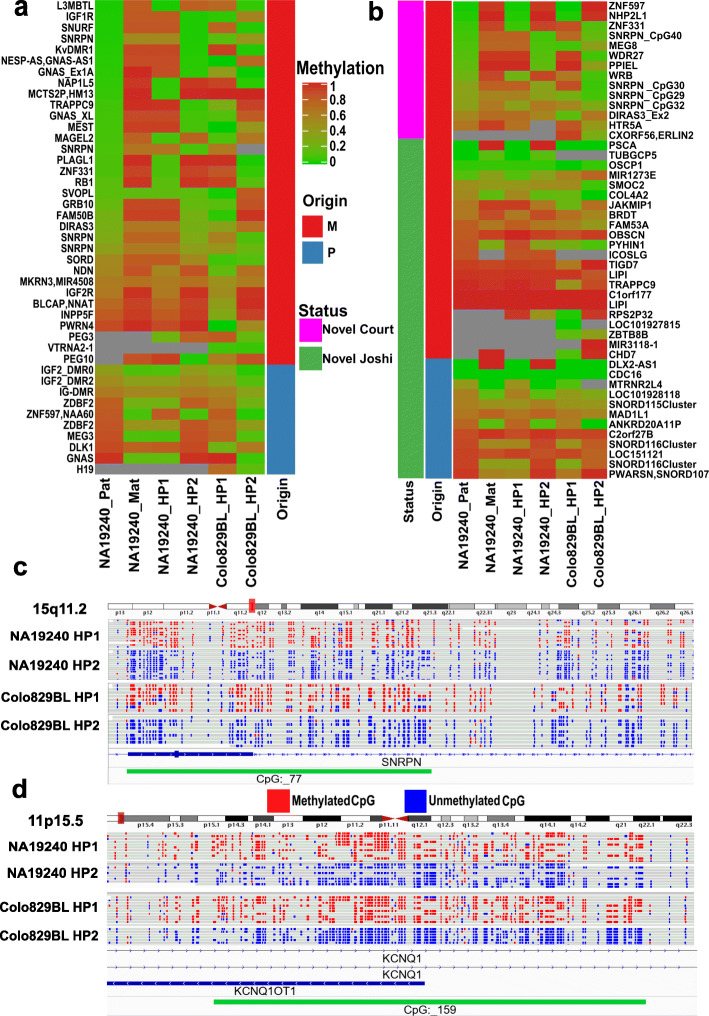
Methylation levels and phased CpGs at human ICRs. **a**, **b** Methylation levels of phased CpGs presented with haplotypes of origin at reported ICRs as heatmaps. **a** CpGs mapped to known ICRs. **b** CpGs mapped to novel ICRs from Court et al. and Joshi et al. The heatmap colors represent the mean of methylation at the regions. Origin bar indicates known or reported origin from previous studies, and heatmap column labels represent assigned haplotype by NanoMethPhase. In trio phasing, Pat stands for paternal and Mat for maternal. **c**, **d** Integrative Genomics Viewer screen captures of phased bam files converted to mock WGBS format for samples NA19240 run 1 and Colo829BL at two well-known ICRs

We used two approaches to evaluate the phasing results. Firstly, we used trio data (parental and child variants) for NA19240 from 1KGP [30] to create a mock phased vcf file to use as input for NanoMethPhase (software code in the repository https://github.com/vahidAK/NanoMethPhase). Out of 4.075M reads assigned to at least one SNV, 1.802M reads assigned to maternal and 1.797M reads to paternal haplotypes (Fig. 3e). 3.464M reads (96.3% of phased reads using trio data and 92.3% of phased reads using nanopore sequencing alone) were congruent between trio and nanopore phasing alone. Trio phasing itself was confirmed by examining the methylation status of paternal and maternal haplotypes at established and putative ICRs. This includes 43 known regions, as well as 14 novel regions from Court et al. [32] and 34 novel regions from Joshi et al. [33] (Additional file 4). As presented in Fig. 4a and b (first two heatmap columns and Additional file 4), NanoMethPhase correctly detected ASM using trio data. The differences in methylation level and correct parental origin is captured at known and novel ICRs. The only obvious inconsistency with the reported parent of origin was *DLX2-AS1* from Joshi et al. but this ICR is only supported by four CpGs. Joshi et al. used a 450k methylation array to study ASM and parent of origin in uniparental disomic subjects [33], and ~ 90% of their reported ICRs are supported by less than seven probes within differentially methylated regions (DMRs). Court et al. used WGBS and high-density methylation microarrays to study ASM [32]. We then compared the phased status of reads from the trio to nanopore sequencing alone. The human genome reference is not completely contiguous, and it is represented by chromosome scaffolds with gaps of unknown sequence (represented by “*N*” in the reference). Moreover, the sequence coverage across the genome is not evenly distributed and there are regions lacking reads. Therefore, when using SNVs from a single sample to phase reads for that sample, we would not expect consistent correct haplotype assignment (i.e., all reads that are from maternal be always on HP1 or HP2 and vice versa). Rather, we would expect reads mapped to a region to group together (e.g., reads that are from maternal haplotype for a given region all being designated either HP1 or HP2 at that region). Therefore, to investigate this, we sampled reads at every 10 kb for trio phasing and nanopore phasing alone and we kept regions with more than two phased reads on one or both haplotypes. We then compared the read haplotype assignment at each 10-kb region to check whether reads that belong to paternal or maternal haplotype at a given position in trio phasing group together as HP1 or HP2 in nanopore phasing alone. We examined 3.3M reads at 236,478 genomic positions. Of these, only 54,592 reads (1.66%) were incorrectly phased in nanopore phasing alone compared to the trio phasing.

Secondly, we investigated the methylation status of phased haplotypes at known ICRs for Colo829BL and NA19240 run 1. In both samples, NanoMethPhase recapitulated methylation differences at known and novel ICRs (Fig. 4a, b; the last four heatmap columns and Additional file 4), although, as mentioned earlier, the haplotype switch is frequent and haplotype assignment is not consistent. We also used Integrative Genomics Viewer [34] to visualize mock bisulfite-converted phased bam files output by NanoMethPhase. This represents accurate ASM at two well-known ICRs (Fig. 4c, d). SNRPN and KvDMR1 aberrant imprinting are involved in Prader-Willi/Angelman and Beckwith–Wiedemann syndromes, respectively [35].

### Differentially methylated regions on chromosome X map to known inactive genes

**Fig. 5:**
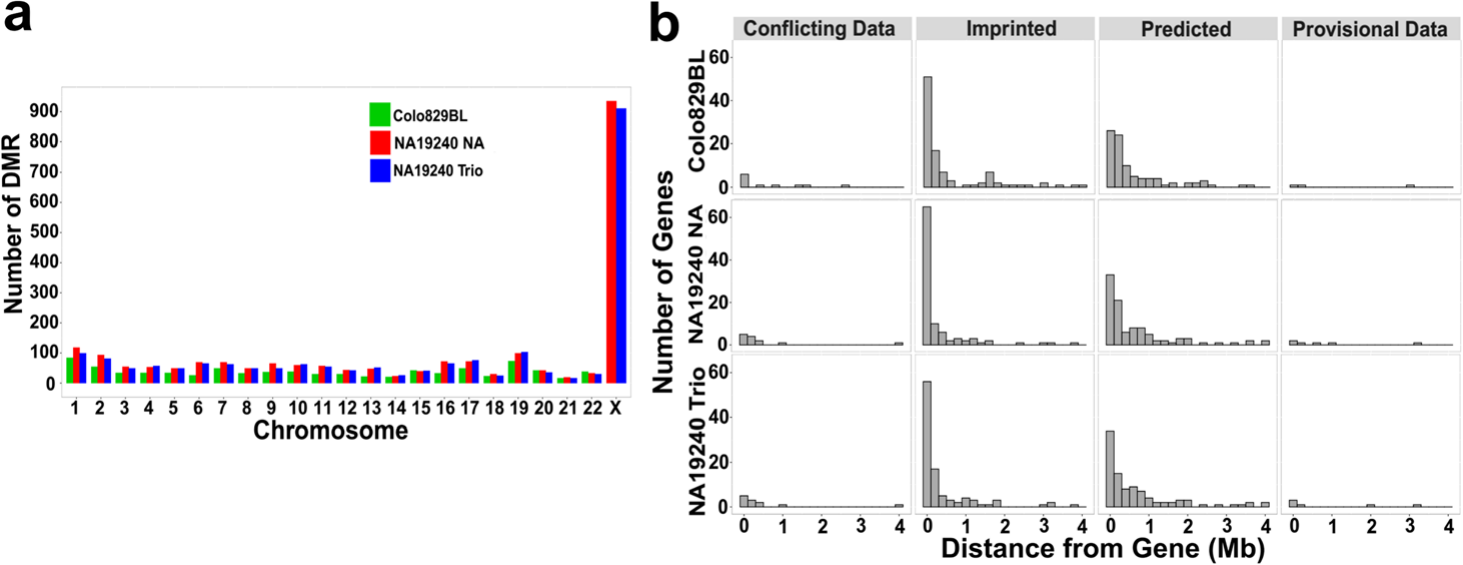
Differentially methylated regions mapping and imprinted genes. **a** Number of DMRs detected at each chromosome in Colo829BL, NA19240 run 1 nanopore alone phasing, and NA19240 run 1 trio phasing. The numerous DMRs in the X chromosome of NA19240 cell line are explained by its X chromosome inactivation. **b** Mapped DMRs to 4 Mb upstream and downstream of known, predicted, conflicting and provisional imprinted genes from GeneImprint and the catalog of human imprinted gene databases. NA19240 NA stands for NA19240 nanopore phasing alone

To investigate DMRs genome-wide, we performed differential methylation analysis (DMA) using the default parameters of the *dma* module in our tool which uses Dispersion Shrinkage for Sequencing data (DSS) [36] R Bioconductor package to found DMRs. We detected 2205 DMRs in NA19240 run 1 nanopore phasing alone and 2109 in trio phasing (Fig. 5a; Additional file 5). Ninety-three percent (1964 DMRs) of DMRs in the trio phasing overlapped with DMRs from nanopore phasing alone. In the male Colo829BL sample, we detected 854 DMRs (Fig. 5a; Additional file 5).

NA19240 is a female B-lymphocyte cell line and therefore presents XCI [3]. Several genes escape XCI (express from both alleles) and they are mostly responsible for sex-specific characteristics in women, while most of the genes are inactivated and display MAE [37]. CpG methylation is an important mechanism through which cells can achieve and maintain XCI. Not surprisingly, ~ 40% of all DMRs in NA19240 mapped to chromosome X. We assessed whether DMRs detected by NanoMethPhase mapped to any escapee genes. For this aim, data from three previous studies [37–39] on genic XCI were collected which includes 371, 204, 75, and 71 inactive, variable, escapee, and unknown genes, respectively (Additional file 6). Given that methylation at the promoter region has a direct effect on gene silencing, we mapped DMRs 1 kb upstream and 200 bp downstream of a TSS. DMRs were mapped to inactive genes in a greater proportion than escapee genes (Table 2; Additional file 7). The presence of methylated CpGs in gene bodies is associated with active genes [40]. Therefore, we would expect to also observe DMRs mapping to the gene body of inactive genes, because only one of the alleles is active. We mapped DMRs to the gene body of the 721 genes. DMRs significantly mapped to inactive genes compared to escapee ones (Table 2; Additional file 7) with the expected methylation direction for genes with DMRs mapped to their promoter and body (i.e., inactive genes with hypermethylation at the promoter mostly displayed hypomethylation at the gene body and vice versa). It is also worth mentioning that the inactive state for the majority of inactive genes with mapped DMR was supported by at least two of the studies, while the majority of escapee genes was supported by one of the studies.

**Table 2:** Mapping DMRs from NA19240 run 1 to genes from chromosome X

Gene category	Sample	Inactive	Escapee	Variable	Unknown	Total*
# of DMRs to GeneBody	NA**	396	16	89	28	519
	Trio	380	17	86	26	503
# of genes with DMR in body	NA	154	9	48	15	226
	Trio	151	10	50	15	226
# of DMRs to promoter	NA	178	8	68	19	264
	Trio	175	9	60	21	257
# of genes with DMR in promoter	NA	190	8	69	19	286
	Trio	187	9	61	20	277
# of genes with DMR in body and promoter	NA	87	2	19	5	113
	Trio	88	2	19	6	115

*All number of unique DMRs or genes.**NA nanopore alone, phasing reads only using nanopore sequencing for a single sample

### Autosomal DMRs mapped to known ICRs and known imprinted genes

We then mapped autosomal DMRs to known and novel ICRs from Court et al. [32] and Joshi et al. [33] (Table 3; Additional file 8). In addition to these ICRs, we mapped DMRs to 166 novel DMRs from Zink et al. [41] from which 83 are on chr15 and 83 are on other chromosomes (Table 3; Additional file 8). Overall, DMRs mapped to 60% of known ICRs and novel ICRs from Court et al. and several novel ICRs from Zink et al. and Joshi et al. (Table 3). Moreover, the parent of origin identified from NA19240 trio phasing for DMRs that mapped to ICRs was consistent with reported parental origin except for *DLX2-AS1* from Joshi et al. (see the section “NanoMethPhase detects allele-specific methylation”) and *RP11-33B1.1* from Zink et al. RP11-33B1.1 was reported with a modest increase in methylation from the maternal allele (0.17) in Zink et al. The consistency in the parent of origin of DMRs further highlights accurate phasing of CpG methylation and parent of origin detection using NanoMethPhase and trio information.

**Table 3:** Mapping DMRs to ICRs

Sample	DMRs mapped	Known	Novel Court	Novel Joshi	Novel Zink
NA19240 NA*	74	28	8	5	24 (6 on chr15)
NA19240 Trio	69	26	8	4	22 (6 on chr15)
Colo829Bl	60	26	10	5	16 (4 on chr15)

*NA nanopore alone, phasing read only using nanopore sequencing for a single sample

Most imprinted genes are located in clusters which can span up to approximately 4 Mb and are controlled by their ICR [42, 43]. Therefore, we investigated the distance of DMRs to known imprinted genes, those predicted to be imprinted, and those genes with conflicting or provisional data. We gathered a list of known, predicted, and conflicting imprinted genes from GeneImprint (http://www.geneimprint.com/) and the catalog of imprinted genes (http://igc.otago.ac.nz/) [44] databases which include 107 imprinted, 103 predicted, 14 conflicting, and 6 provisional (Additional file 9) genes. We mapped DMRs to regions spanning 4 Mb upstream and downstream of these genes (Fig. 5b; Additional file 10). Fifty percent of autosomal DMRs in NA19240 run 1 and 45% in Colo829BL mapped to this window. Eighty percent of known imprinted genes in NA19240 run 1 and 74% in Colo829BL had at least one DMR mapped within 1 Mb from the gene boundaries.

The best correlation between nanopore CpG methylation calls with WGBS was obtained at regions with 4–8% of CpG ratio (number of CpGs/region length; Additional file 2: Fig. S1g). We noticed that the known ICRs with CpG ratio 4–8 tend to be more detected. Overall, 28 of the known ICRs have CpG ratio 4–8% and 15 less than 4% or more than 8%. In Colo829BL, 26 known ICRs were detected from which 21 have 4–8% CpG ratio. In NA19240, out of 28 detected known ICRs, 21 have 4–8% CpG ratio. However, in the HG002 sample (~50x. Ashkenazi son; see below and Additional file 1: Section 5), which has higher coverage compare to NA19240 run 1 and Colo829BL, out of 38 detected known ICRs, 26 have 4–8% CpG ratio.

As ONT frequently releases new versions of Guppy with claimed higher accuracy, we aimed to investigate the effect of a new version of the basecaller on ASM detection (Additional file 1: Section 4). We re-basecalled Colo829BL and NA19240 run 1 using guppy v4.2.2. Even though more true-positive SNVs were detected at the optimal threshold, more false positives were also presented (Table 1; Additional file 1: Table S2). Therefore, no overall improvement in the SNV detection was observed. However, using guppy v4.2.2, the detection of ICRs slightly improved and we could detect one more reported ICR [32, 33, 41] in NA19240 and 6 more in Colo829BL (Table 3; Additional file 1: Table S3) and, on average, about 82% of detected DMRs in Guppy 4.2.2 were overlapped with DMRs detected with older Guppy.

In NA19240 trio phasing, the SNV calls are obtained from short-read data. To further investigate ASM detection in trio where SNV calls originating from nanopore sequencing, we used nanopore data for GIAB Ashkenazi trio [29] samples including son (HG002), father (HG003), and mother (HG004) (Additional file 1: Section 5). This analysis demonstrated the detection of most of the known ICRs (86%) (Additional file 1: Table S4). Moreover, detected DMRs and phased reads in phasing using SNVs detected from nanopore data for all samples in trio demonstrated high commonalities with DMRs and phased reads for the trio and phasing using SNVs detected from short-read sequencing (high confidence variant calls, obtained from short-read sequencing, for Ashkenazi trio is also available in GIAB).

## Discussion

ASM is involved in various processes such as development and tissue differentiation. Its dysregulation can result in developmental disorders and promotes cancer [2, 5, 6]. Short-read sequencing coupled with bisulfite treatment is used for the detection of ASM. However, the length of reads and the complexity and biases introduced by bisulfite treatment can impede their investigation, especially in low SNP density regions. In these regions, the length of reads can be insufficient to span multiple SNPs preventing their association in a contiguous haplotype. This conceptual limit is not shared by long-read sequencing via nanopore. The span of reads reduces the risk of them not reaching enough SNP positions. It also enables the filtering of low-quality SNPs, reducing the presence of erroneous SNPs with a minimal impact on the number of reads that spans multiple SNPs. Long-read sequencing powered by ONT is also applicable to detect mono-allelic methylation as it offers detection of both DNA bases and their modifications. Mono-allelic bisulfite-converted C are indistinguishable from a C to T SNP present in the sample using WGBS alone [8]. The signal from the pore can be used to get the sequence confirmation that would otherwise require another input for SNP presents in the sample, a feature that can also be leveraged to investigate cancers presenting a loss of heterozygosity [45].

In this study, we benchmarked methylation calling tools for nanopore sequence data, improved SNV calling for lower coverage data, and detected ASM. We developed the required tools, SNVoter and NanoMethPhase, which allow users to detect ASM using a command-line interface. We demonstrated that nanopore methylation calls are concordant with gold standard platforms (Fig. 1; Additional file 2: Fig. S1) and this technology is advantageous in highly repetitive regions, particularly at highly repetitive ALR/Alpha satellite (Additional file 2: Fig. S2) which their expression is shown to be enriched in cancer [46]. A drawback of nanopore sequencing is the high cost which increases dramatically when several flow cells need to be run to obtained adequate coverage. We determined that our workflow is capable of detecting ASM using low coverage (~ 10×) of nanopore data from a single PromethION flowcell run (one PromethION run typically provides a coverage of 10–25× using the r9.4 pore). Therefore, the usage of NanoMethPhase is advised to leverage the depth of the long-read data. We demonstrated detection of ASM and parent of origin using trio information (Fig. 4). We were able to detect ASM by nanopore sequencing exclusively from a single sample which was concordant with trio phasing, although the parent of origin labeling is inconsistent between non-overlapping haplotyped regions (Fig. 4). 

Mnimap2 [47] is a widely used aligner for nanopore long-read data; therefore, we also investigated the possibility of improvement in SNV detection using the recently developed aligner for nanopore long reads, Winnowmap [48]. We compared SNV detection for Colo829BL aligned to hg38 using Winnowmap to Minimap2 and no improvement was observed (Additional file 2: Fig. S8).

ASMs on chromosome X were detected by NanoMethPhase in the NA19240 cell line. They mostly mapped to known inactive genes on the chromosome X (Table 2; Additional file 7). Previous studies mostly relied on transcriptome analysis to investigate genic XCI [37–39], but this provides indirect evidence of the inactivation rather than detecting the actual mechanism of inactivation. Our approach can be applied, along with expression-based approaches, to further investigate genic XCI and CpG methylation as a potential mechanism for gene inactivation. We indicated that autosomal DMRs detected by NanoMethPhase mapped to most of the known ICRs and several other novel ICRs from previous studies [32, 33, 41] (Fig. 4 and Table 3, Additional file 4 and Additional file 8). The majority of known imprinted genes were also detected with one or more autosomal DMR mapped inside or in close vicinity to the gene (Fig. 5; Additional file 10). Numerous autosomal DMRs, which did not map to any known ICRs, were mapped to the close vicinity of the known imprinted genes (Additional file 10). This is also observed by previous studies [21, 41], and these DMRs could be secondary ICRs (also known as somatic DMRs) which usually regulated by nearby germline or primary ICRs and established postfertilization [21, 41, 49]. In addition to imprinting, a proportion of ASM might result in random MAE. Approximately 22% of autosomal DMRs from NA19240 and 18% from Colo829BL mapped to the gene body or the promoter of 251 and 143 MAE genes from Savova et al., respectively [50] (Additional file 11).

We noticed more than 90% of DMRs mapped to ICRs and promoters of genes on chromosome X and more than 80% of DMRs that mapped near imprinted genes had area statistics (the sum of the test statistics of all CpG sites within the DMR) ≥ |100|, while approximately 40% of DMRs in each sample had area statistics < |100|. This is consistent with the fact that DMRs with higher area statistics are more likely to be true positives, and demonstrates the positive impact of filtering the numerous detected DMRs. Several regions were lacking high-quality mapped reads in the original alignment file and phased alignment results. For example, five to seven of known and novel ICRs reported by Court et al. [32] and Joshi et al. [33] lacked mapped reads in the original alignment file and/or the phased alignment results (Fig. 4a, b; Additional file 4). Moreover, there were several ICRs with moderate differences in methylation between haplotypes. For example, approximately 15 to 18 ICRs showed 0.1–0.3 delta in mean methylation, while none of them was captured by the DMA. The Nanopolish algorithm assigns the same log-likelihood ratio to all CpGs in close vicinity of less than 11 bp. Therefore, improvements in methylation calling can improve ASM detection, specifically at dense CpG sites. Although final methylation frequencies obtained by nanopore are similar to those from WGBS and tools that were developed for WGBS DMA are expected to be applicable to nanopore, it is advantageous to have dedicated software to perform DMA from nanopore data. As discussed by Gigante et al. [21], WGBS calls are binary while nanopore tools output continuous predictions or log-likelihood ratios. Algorithms that could leverage non-discrete data present an opportunity to improve DMA. We also noticed that averaging CpG methylation in genomic bins significantly improves correlation with WGBS, even when disregarding the coverage filters (Additional file 2: Fig. S5). This suggests that a sliding window approach might be beneficial for nanopore DMA.

## Materials and methods

### Nanopore sequencing and datasets

Nanopore sequencing data for NA19240 [26], NA12878 [24], and Ashkenazi trio [29] human cell lines are publicly available. A complete description of the datasets, their base calling, mapping, and usage in our study are provided in additional file 1 along with the link to the sources.

We also sequenced the Colo829BL B-lymphoblast cell line using one nanopore PromethION flow cell and Illumina paired-end sequencing at 30× coverage. A complete description of nanopore and Illumina sequencing protocols and data obtained is also provided in Additional file 1.

### CpG methylation calling from nanopore data

To call CpG methylation, we benchmarked three model-based approaches: Nanopolish [10], Megalodon [14], and DeepSignal [15]. Nanopolish uses a hidden Markov model to call CpG methylations from raw nanopore data while Megalodon and DeepSignal use neural networks. We called CpG methylation using these tools (with the default parameters) for 12 flow cells of NA12878 publicly available data (Additional file 1) and compared the results with WGBS data from ENCODE project (ENCFF835NTC) [51] and Human Methylation 27 (27k) array from Fraser et al. [25].

### Variant calling

We used Clair to call SNVs [22]. We called variants for each chromosome using *clair.py callVarBam --threshold 0.2* and the *HG122HD34* model. Indels were filtered out. To evaluate variant calling, we compared SNVs called by Clair from nanopore data to those from 1KGP phase 3 [30] (GRCh37 coordinates). Clair’s variant calls were lifted over to GRCh37 human reference genome coordinates using CrossMap [52] for comparison to 1KGP data.

For our in-house Colo829BL sample, we compared Clair variant calls to Strelka [53] v 2.9.10 calls made from paired-end Illumina reads (Additional file 1).

### Model training to improve SNV calling

We calculated average qualities and mutation frequencies for each position of each 5-mer window containing an SNV. Mutation frequencies were calculated as the number of instances over coverage for each genomic position in the 5-mer window. Base qualities for a given position were calculated as the average of all base qualities mapped to the position. We used these as inputs to a fully connected artificial neural network classifier composed of four hidden layers with a relu activation function. The first hidden layer is six times larger than the input layer and the size of subsequent hidden layers decreases through a factor two.

We trained three models to compare the classifier using different coverages. NA12878 20 flow cells (24×), NA12878 all flow cells (44×), and HG003 (80×) were used for training. First, we called variants for each dataset using Clair and then determined true and false positives using high-quality variants using the Genome in a Bottle database (GIAB) [27]. Using NA12878 20 flow cell data, a randomly selected balanced dataset of 25 million 5-mers was used for training and 4 million unseen randomly selected 5-mers were used as the validation set. For the NA12878 whole dataset and HG003 sample, the training datasets were 18M and 14.9M, respectively, and validation sets were 2.5M and 2M, respectively (Additional file 2: Fig. S6). The NA12878 20 flow cell model was used for < 30× coverage data, NA12878 all flow cells for 30×–45× coverage data, and HG003 model for > 45 coverage data.

### Phasing single nucleotide variants detected from nanopore sequencing

In order to phase nanopore reads and CpG methylation, we first called SNVs for both samples (NA19240 run 1 and Colo829BL) using Clair [22], then used SNVoter to normalize the quality scores and filter out false positives (Fig. 2e and Table 1). Finally, we used WhatsHap [23, 31] v0.18 with the default parameters and *--ignore-read-groups* on to determine haplotype status for each SNV.

### Phasing of nanopore reads and CpG methylations

Phased SNVs and CpG methylation calls were leveraged to phase reads along their CpG methylation to diploid haplotypes. After filtering out a considerable number of false-positive SNVs using SNVoter, we still noticed 10–20% false-positive SNV calls in the datasets (Table 1). These unfiltered false-positive calls, in addition to sequencing errors, can result in reads incorrectly mapping to the SNVs from haplotype 1 when the read would actually belong to the haplotype 2 and vice versa. We noticed reads presenting SNVs from both haplotypes when mapping them to phased SNVs. In NA19240 run 0, out of ~ 3M reads which mapped to at least one phased SNV, ~ 2M reads had SNVs from both haplotypes (Additional file 2: Fig. S7a). To further overcome false positives and the sequencing error problem, we made several filtering steps to account for remnant false-positive SNVs and haplotype ratio (number of SNVs from HP1/HP2 or HP2/HP1). As we analyzed NA19240 run 0, we noticed a lower base quality distribution for false-positive SNVs compared to true positives that could not be filtered out by SNVoter (Additional file 2: Fig. S7b). Therefore, we assigned a minimum base quality threshold to successfully map each read at a phased SNV position. To manage reads containing SNVs from both haplotypes, we defined another threshold, the haplotype ratio, which ensures the reads are assigned to a single haplotype. Based on the quality distribution of SNVs (Additional file 2: Fig. S7b), the proportion of false positives which is between 10 and 20% (Table 1) and haplotype ratios (Additional file 2: Fig. S7a), and also based on empirical phasing at a few known imprinted regions, we used seven as the minimum base quality and 0.75 as haplotype ratio. We also used two as the minimum number of phased SNVs a read must present to be considered for phasing. In order to assign a read to a defined haplotype, a read must satisfy the following criteria:

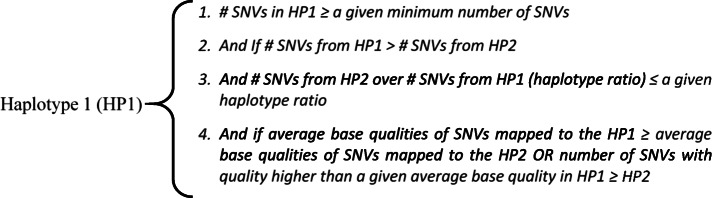



As the reads are separated to different haplotypes, their associated CpG methylations from processed methylation call file are also separated to the corresponding haplotypes. We have integrated all the steps and filters in our python3 command-line tool, NanoMethPhase. Users can input methylation call data from Nanopolish, phased variant calling file, alignment file, and reference genome to NanoMethPhase (Fig. 3c). NanoMethPhase will output phased reads in aligned format, phased mock WGBS converted format for visualization (see the “Visualization” section; Fig. 4c, d), phased methylation calls, and methylation frequency files. The latter can be used for differential methylation analysis to detect DMRs between haplotypes.

### Differential methylation analysis

After phasing reads and CpG methylation to haplotypes, NanoMethPhase can perform DMA to detect mono-allelic methylated regions. It uses the DSS R package [36] for DMA. Users can perform all analyses in a command-line interface and directly perform DMA using the *dma* module of NanoMethPhase on the output phased methylation frequency data to detect DMRs.

### Visualization

NanoMethPhase can convert phased reads into separate mock-WGBS bam files using the processed methylation call file from its *methyl_call_processor* module. Each cytosine in each CpG in each read is converted to a T, A, or N depending on the CpG being called as methylated, unmethylated, or uncalled. These pairs of files can be loaded into a genome browser such as IGV [34] in bisulfite mode for visualization (Fig. 4c, d).

## Supplementary Information

**Additional file 1.** This file includes additional notes for material and methods section. Description of datasets and the sources of publically available data. The results of further analyses using newer version of Guppy basecaller and Ashkenazi trio are also provided in this file.

**Additional file 2.** Contains additional figures for the paper, such as more correlation analysis for comparison of nanopore methylation call with WGBS and mapping of nanopore specific CpG methylation calls to genomic regions, supporting figures for SNV improvement using SNVoter, Read length distribution for Colo829BL and NA19240, Model training plots for SNVoter, etc.

**Additional file 3.** Contains table of true- and false-positives using different quality thresholds for Clair and Clair+SNVoter.

**Additional file 4.** Contains average of methylation at known and novel ICRs from Court et al. and Joshi et al.

**Additional file 5.** Contains DMRs found in each sample.

**Additional file 6.** List of genes studied by Carrel and Willard, Cotton et al., and Tukiainen et al. for genic XCI.

**Additional file 7.** Mapping of DMRs on chromosome X for NA19240 run 1 to the list of genes studied through XCI.

**Additional file 8.** Mapping of autosomal DMRs to known and novel ICRs.

**Additional file 9.** List of imprinted, predicted, conflicting, and provisional imprinted genes from GeneImprint (http://www.geneimprint.com/) and the catalog of human imprinted gene (http://igc.otago.ac.nz/home.html) database.

**Additional file 10.** Mapping DMRs to 4 Mb upstream and downstream of the list of imprinted, predicted, conflicting, and provisional imprinted genes.

**Additional file 11.** Mapping of DMRs to the reported MAE genes by Savova et al.

**Additional file 12.** Review history.

## Data Availability

NanoMethPhase and SNVoter source codes, installation instructions, and tutorials are available on GitHub [54, 55] (https://github.com/vahidAK/NanoMethPhase and https://github.com/vahidAK/SNVoter) under GNU General Public License v3.0 (https://www.gnu.org/licenses/). Source codes for NanoMethPhase and SNVoter are also deposited in Zenodo as DOI-assigned repositories [56, 57]. Illumina sequencing alignment file and nanopore raw fast5 and basecalled fastq files for the Colo829BL sample are available at the European Genome-phenome Archive (EGA: https://www.ebi.ac.uk/ega/home) under the accession number EGAS00001001385 [58]. NA19240 data from De Coster et al. [26] are publically available at ENA (https://www.ebi.ac.uk/ena/browser/home) under the accession number PRJEB26791. Nanopore data for NA12878 from Jain et al. [24] are publically available as an Amazon Web Services Open Data set at https://github.com/nanopore-wgs-consortium/NA12878. Ashkenazi trio data (HG002, HG003, and HG004) from Zook et al. [29] are publically available at SRA (https://www.ncbi.nlm.nih.gov/sra) under the accession number PRJNA200694 and Genome in a Bottle GitHub (https://github.com/genome-in-a-bottle/giab_data_indexes).
