# Supplementary material for: Megabase-scale methylation phasing using nanopore long reads and NanoMethPhase
Source: Genome Biol. 2021 Feb 22;22:68. doi: 10.1186/s13059-021-02283-5 (PMC7898412; doi:10.1186/s13059-021-02283-5)
Supplement: Supplementary file 1 — Additional file 1. This file includes additional notes for material and methods section. Description of datasets and the sources of publically available data. The results of further analyses using newer version of Guppy basecaller and Ashkenazi trio are also provided in this file. [file 13059_2021_2283_MOESM1_ESM.docx]

**Additional File 1**

Table of Contents

[1 Datasets used in our Study 2](#_Toc63593432)

[1.1 Public Datasets 2](#_Toc63593433)

[1.1.1 NA12878/GM12878 Native DNA: 2](#_Toc63593434)

[1.1.2 HG003: 3](#_Toc63593435)

[1.1.3 NA19240/GM19240: 3](#_Toc63593436)

[1.2 Colo829BL 3](#_Toc63593437)

[1.2.1 DNA Extraction and Nanopore Sequencing 3](#_Toc63593438)

[1.2.2 DNA Extraction and Illumina Sequencing 4](#_Toc63593439)

[2 Variant Calling 6](#_Toc63593440)

[2.1 From Nanopore Data 6](#_Toc63593441)

[2.2 From Illumina for Colo829BL 6](#_Toc63593442)

[3 Phasing 6](#_Toc63593443)

[3.1 Trio Phasing for NA19240 Run 1 6](#_Toc63593444)

[3.2 Phasing NA19240 Run 1 and Colo829BL Using Nanopore Sequencing Alone 7](#_Toc63593445)

[4 Detecting ASM using Guppy v4.2.2 7](#_Toc63593446)

[5 Ashkenazi Trio 8](#_Toc63593447)

[6 References 9](#_Toc63593448)

# Datasets used in our Study

## Public Datasets

### NA12878/GM12878 Native DNA:

NA12878 human genome reference standard is sequenced on 53 MinION R9.4 chemistry (FLO-MIN106) by Oxford Nanopore Technologies (ONT) [1]. NA12878 rel6 dataset (<https://github.com/nanopore-wgs-consortium/NA12878/>) served as training set to develop our SNV classifier (SNVoter). Moreover this dataset was used to investigate the reliability of CpG methylation calling by Nanopore sequencing and to benchmark three CpG methylation calling tools for nanopore sequencing; Nanopolish [2], DeepSignal [3], and Megalodon [4]. We used raw Nanopore sequencing data from 12 flow cells (FC) (Table S1) to investigate methylation calling to benchmark three mentioned tools. Moreover, 13 other FCs of NA12878 along with 7 FCs from methylation calling (Table S1) used for training purpose for our SNV classifier for data with coverage <30x (20 FCs gave us ~24x coverage. Fig. S1). The raw fast5 files from these flow cells base called in-house using Guppy v3.4.4 and 450bps high accuracy model. We also used the 20 flow cells plus other flow cells based called by ONT using guppy v 2.3.8 flipflop model to train a model for data with higher coverage (All flow cells, 44x coverage. Fig. S1). Minimap2 [5] and SAMtools [6] were used to map, sort and index base called reads to the GRCh38 human genome reference (<https://genome.ucsc.edu/>) via the following command:

Table S1: Flow cells from NA12878 dataset used for methylation calling and training low coverage model.

| Flow cells used for methylation | | | 20 Flow cells used for model training | | | | | |
| --- | --- | --- | --- | --- | --- | --- | --- | --- |
| Flow Cell | **Kit** | **Pore** | **Flow Cell** | **Kit** | **Pore** | **Flow Cell** | **Kit** | **Pore** |
| FAF09968 | Ultra | R9.4 | **FAF01169** | Ligation | R9.4 | **FAB49164** | Ligation | R9.4 |
| FAF09277 | Ultra | R9.4 | **FAF01441** | Ligation | R9.4 | **FAF05869** | Ligation | R9.4 |
| FAF15586 | Ultra | R9.4 | **FAB45277** | Ligation | R9.4 | **FAB39088*** | Ligation | R9.4 |
| FAB42473 | Ligation | R9.4 | **FAF01127** | Ligation | R9.4 | **FAB39075*** | Ligation | R9.4 |
| FAB39088* | Ligation | R9.4 | **FAF01132*** | Ligation | R9.4 | **FAB42316*** | Ligation | R9.4 |
| FAB39075* | Ligation | R9.4 | **FAB49712** | Ligation | R9.4 | **FAB44989*** | Ligation | R9.4 |
| FAB42316* | Ligation | R9.4 | **FAF01253** | Ligation | R9.4 | **FAB45332*** | Ligation | R9.4 |
| FAB42451* | Ligation | R9.4 | **FAB49914** | Ligation | R9.4 | **FAB43577** | Ligation | R9.4 |
| FAB45332* | Ligation | R9.4 | **FAB42451*** | Ligation | R9.4 | **FAB42476** | Ligation | R9.4 |
| FAB44989* | Ligation | R9.4 | **FAB45271** | Ligation | R9.4 | **FAB39043** | Ligation | R9.4 |
| FAF01169 | Ligation | R9.4 | *These flow cells were used for both methylation calling and training | | | | | |
| FAF01132* | Ligation | R9.4 |  |  |  |  |  |  |

### HG003:

This data set which provided by GIAB [7] was used to train a model for high coverage data. Base called reads using guppy v 3.2.5 flipflop model were downloaded from GIAB (<ftp://ftp-trace.ncbi.nlm.nih.gov/giab/ftp/data/AshkenazimTrio/HG003_NA24149_father/UCSC_Ultralong_OxfordNanopore_Promethion/>). Finally reads were mapped, sort, and index using minimap2 and samtools as explained in 1.1.1 section. Fig. S1 represents the coverage for final alignment file for this sample. We got a coverage 0f ~80x from this sample.

### NA19240/GM19240:

This dataset were adapted from Wouter De Coster and colleagues [8]. They have sequenced NA19240 standard DNA on 5 PromethION using 1D DNA ligation sequencing kit SQK-LSK109 and SQK-LSK108 [8]. We used this data set to comprehensively investigate the performance of our classifier (SNVoter) and to phase nanopore reads and DNA methylation. NA19240 run 1 (ERR3046935 or ERX3247335 or ERR3219854) was used for CpG methylation detection and phasing purposes. Run 1 raw sequencing data base called in-house using Guppy v3.2.2 and 450bp high accuracy model. Moreover, to test our classifier at various coverages we also used base called fastq files (Guppy v2.2.3 flipflop model) from other NA19240 runs (Runs 0, 2, 3, and 4). Runs 0 (ERR3219853), 1 (ERR3219854), 1 and 2 (ERR3219854-5), 0 and 1 (ERR3219853-4), and 1-4 (ERR3219854-7) were used to test SNV classifier at ~18x, ~22x, ~32x, ~40x, and ~65x, respectively (Fig. S1). We used minimap2 and samtools as explained in 1.1.1 section to map reads to the GRCh38 human genome reference, sort, and index the alignment files.

## Colo829BL

### DNA Extraction and Nanopore Sequencing

High molecular weight (HMW) DNA was extracted using the Qiagen MagAttract HMW DNA Kit (cat. no. 67563, QIAGEN, Germantown, MD, USA). Genomic libraries were prepared, conforming to Oxford Nanopore Technologies’ protocols, using the SQK-LSK109 Ligation Library Kit. NEB Ultra II kit (New England Biolabs, Ipswich, MA, USA, cat. no. E7646A) was used for end-repair and A-tailing. NEBNext quick ligase (E6056S) was used to ligate the Oxford Nanopore adapters. A final size selection of 0.4:1 ratio (magnetic beads to library) was done to select against smaller molecules. PromethION sequencing proceeded using the R9.4.1 pore flow cell on the PromethION Alpha-Beta instrument and the beta release software version 19.06.9 (MinKNOW v3.4.6, GUI v3.4.12). A DNase I nuclease flush (Invitrogen cat no. AM2222, INSERT BUFFER FORMULA HERE) was performed after 18 hours as per Oxford Nanopore protocol, version NFL_9076_v109_revD_08Oct2048.

Output fast5 files were base called using guppy v3.2.1. Base called reads were mapped to the GRCh38 reference genome, sorted and indexed using minimap2 (section 1.1.1).

### DNA Extraction and Illumina Sequencing

Co-extraction of DNA and RNA from cell pellet was performed using an ALine EvoPure kit (Aline Biosciences, R-907-400-C5) automated on MicroLab NIMBUS (Hamilton) liquid handling robot. To minimize library bias and coverage gaps associated with PCR amplification of high GC or AT-rich regions, a version of the TruSeq DNA PCR-free kit (E6875-6877B-GSC, New England Biolabs), automated on a Microlab NIMBUS liquid handling robot (Hamilton) was employed. Briefly, 500ng of genomic DNA was arrayed into wells in a 96-well microtitre plate and subjected to shearing by sonication (Covaris LE220). Sheared DNA was end-repaired and size-selected using paramagnetic PCRClean DX beads (C-1003-450, Aline Biosciences), targeting a 350-450 bp size range.After 3’ A-tailing, full length TruSeq adapters were ligated to DNA fragments. Libraries were purified using paramagnetic (Aline Biosciences) beads. Prior to sequencing, PCR-free genome library concentrations were quantified using a qPCR Library Quantification kit (KAPA, KK4824).

Library was sequencened on Illumina HiseqX using v2.5 reagents and paired end 150 base reads according to manufacturer’s protocols.

Illumina paired-end sequencing data were mapped to the GRCh38 using BWA MEM [9] and *bwa mem –M* setting.


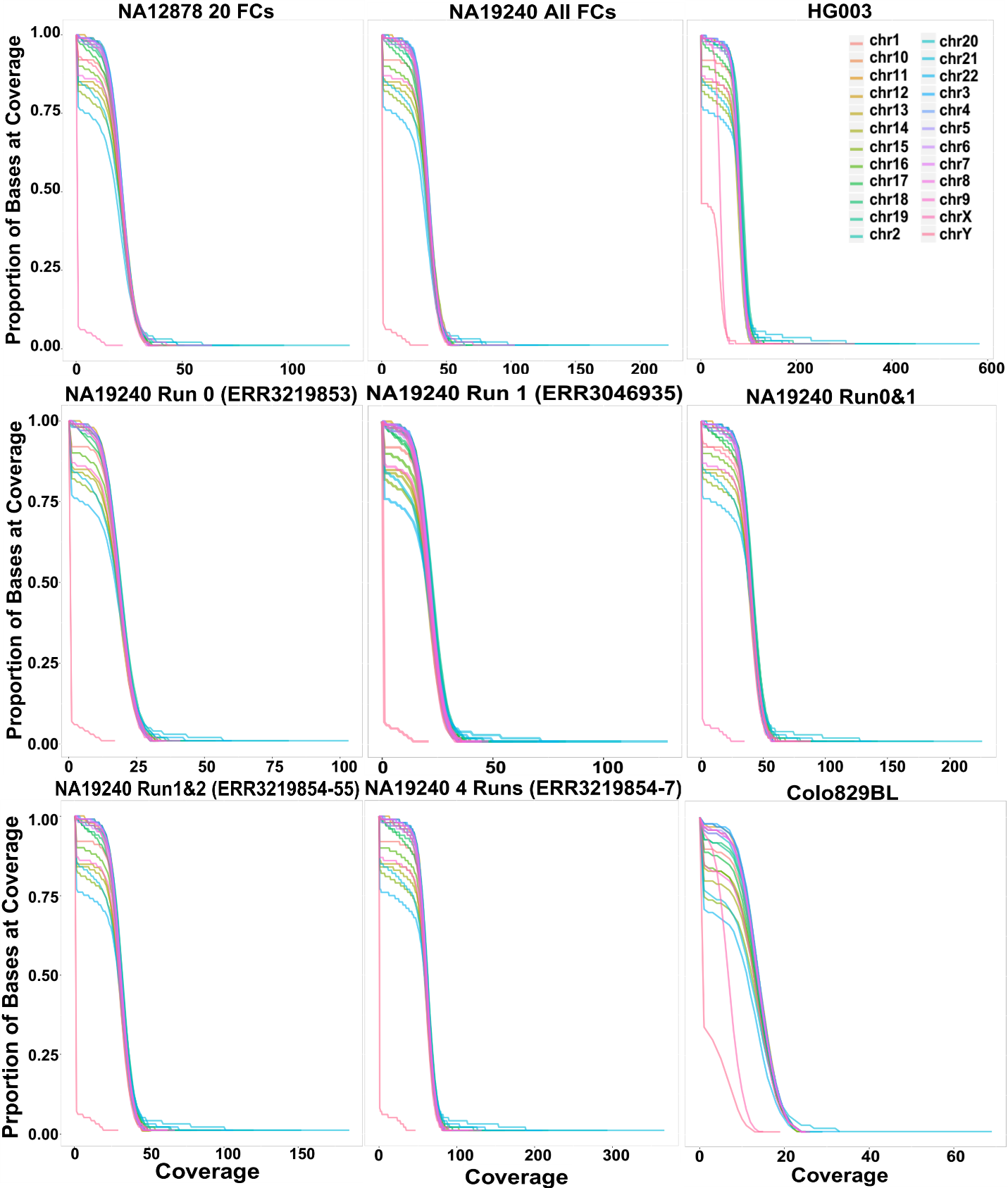


Fig. S1: Coverage distribution of different samples used in our study.

# Variant Calling

## From Nanopore Data

For each dataset we used Clair [10] to call variants for each chromosome using the following command:

Finally all variant call files were merged to build genome-wide variant calls.

## From Illumina for Colo829BL

We used Strelka v 2.9.10 [11] to call variants from Colo829BL paired end sequencing data. We first build the running work flow for Strelka using the following command:

We then ran the workflow created by Strelka to call variants for Colo829BL sample. Finally, high-quality passed SNVs from Strelka were extracted.

# Phasing

## Trio Phasing for NA19240 Run 1

For phasing NA19240 run 1 reads and CpG methylations using maternal and paternal SNVs, we used phase 3 variant call data for mother (NA19238), father (NA19239) and child (NA19240) from 1000 genome project [12] phase3. We made a mock phased vcf file using trio vcf data via the script *Trio_To_PhaseVCF_4FemaleChild.sh* in the github repository (<https://github.com/vahidAK/NanoMethPhase>). Phase 3 variant calls are in GRCh37 coordinates. Therefore, CrossMap [13] was used to convert GRCh37 coordinates to GRCh38. Finally NanoMethPhase was used to phase methylome:

## Phasing NA19240 Run 1 and Colo829BL Using Nanopore Sequencing Alone

After calling SNVs for these samples using Clair (see section 2.1), we used SNVoter to normalize Clair variant call qualities for NA19240 run 1 and Colo829bl samples.

We used 350 as the quality threshold to filter out low quality normalized calls from NA19240 and for Colo829BL we used 160 as the quality threshold (Table 1 in the paper). We then used WhatsHap v 0.18 [14] to phase these SNVs using the following command:

Finally, NanoMethPhase was used to phase methylome for each sample:

# Detecting ASM using Guppy v4.2.2

Oxford Nanopore Technologies constantly releases new base caller versions. To investigate if using newer version of base caller improves ASM detection we re-basecalled Colo829BL and NA19240 samples using Guppy v4.2.2.

Variant calling using Clair and then improving Clair’s calls using SNVoter resulted in detection more true positive SNVs but also more false positive and only improved recall (Table S2). Using the new version

Table S2: Different metrics for SNV calls using Clair and Clair + SNVoter and newest guppy version.

| Tool | Sample | QT | All TP | TP | FP | Acc | Pre | Rec | F1 |
| --- | --- | --- | --- | --- | --- | --- | --- | --- | --- |
| C | Colo829BL | 725 | 3638416 | 2867820 | 968430 | 0.88 | 0.77 | 0.83 | 0.79 |
| C+S | Colo829BL | 160 | 3638416 | 2946434 | 467195 | 0.91 | 0.86 | 0.86 | 0.86 |
| C | NA19240 Run1 | 750 | 3973249 | 3577947 | 1035519 | 0.82 | 0.78 | 0.94 | 0.85 |
| C+S | NA19240 Run1 | 310 | 3973249 | 3582691 | 742506 | 0.87 | 0.83 | 0.94 | 0.88 |

After methylation calling and phasing, DMA between haplotypes was performed. 876 (~77% in common with base calling using old guppy) DMRs detected in Colo829BL, 2090 (~89% in common with base calling using old guppy) in NA19240 pure nanopore, and 1965 (~82% in common with base calling using old guppy) in NA19240 trio phasing. We mapped detected DMRs to known and novel DMRs reported by three previous studies [15–17] (Table S3). This resulted in detection of very few more reported ICRs.

**Table S3**: Mapping DMRs to reported known and novel ICRs.

| Sample | DMRs Mapped | Known | Novel Court | Novel Joshi | Novel Zink |
| --- | --- | --- | --- | --- | --- |
| NA19240 NA* | 76 | 28 | 9 | 5 | 24 (6 on chr15) |
| NA19240 Trio | 70 | 25 | 9 | 4 | 23 (6 on chr15) |
| Colo829Bl | 66 | 28 | 9 | 6 | 20 (5 on chr15) |

*NA: Nanopore alone, phasing read only using nanopore sequencing for a single sample.

# Ashkenazi Trio

Ashkenazi trio base called reads for all three samples along with promethION raw fast5 files for HG002 were downloaded from GIAB GitHub and GIAB BioProject in SRA (<https://github.com/genome-in-a-bottle/giab_data_indexes> and PRJNA200694). Ahkenazi trio has base called nanopore sequencing data publically available. Moreover, there are publically available variant call data detected using Illumina sort-read sequencing for this trio.

We phased reads in son (HG002) in two ways. First, where all SNVs in trio detected from nanopore data via Clair (SNVoter was not used as the data have high coverage). Second, where SNVs for the trio are coming from publically available variant call data detected using sort-read sequencing.

In phasing using SNVs detected from nanopore data for the trio, 1.62M reads were assigned to the maternal and 1.62M reads to the paternal haplotype and in phasing using SNVs from GIAB database 1.44M reads were assigned to the maternal and 1.44M reads to the paternal haplotype (1.38M reads were in common in maternal and 1.38M in paternal).

We detected 1,812 DMRs when reads were phased using GIAB variant call files and 2,066 when phased using variant detected from nanopore in trio (1,726 were overlapped). DMRs were mapped to known and novel ICRs reported by the previous studies [15–17] (Table S4). They also mapped to 4Mb window around known imprinted genes (GeneImprint <http://www.geneimprint.com/> and the catalogue of imprinted genes <http://igc.otago.ac.nz/>) (Fig. S2).

**Table S4** : Mapping DMRs to reported known and novel ICRs.

| Sample | DMRs Mapped | Known | Novel Court | Novel Joshi | Novel Zink |
| --- | --- | --- | --- | --- | --- |
| HG002 GIAB* | 82 | 37 | 10 | 7 | 23 (1 on chr15) |
| HG002 Nanopore** | 87 | 38 | 11 | 9 | 23 (1 on chr15) |

*Reads were phased using high confidence GIAB variant call file which stem from short read sequencing. ** Reads were phased using SNVs detected from nanopore sequencing in all three sample in the trio.


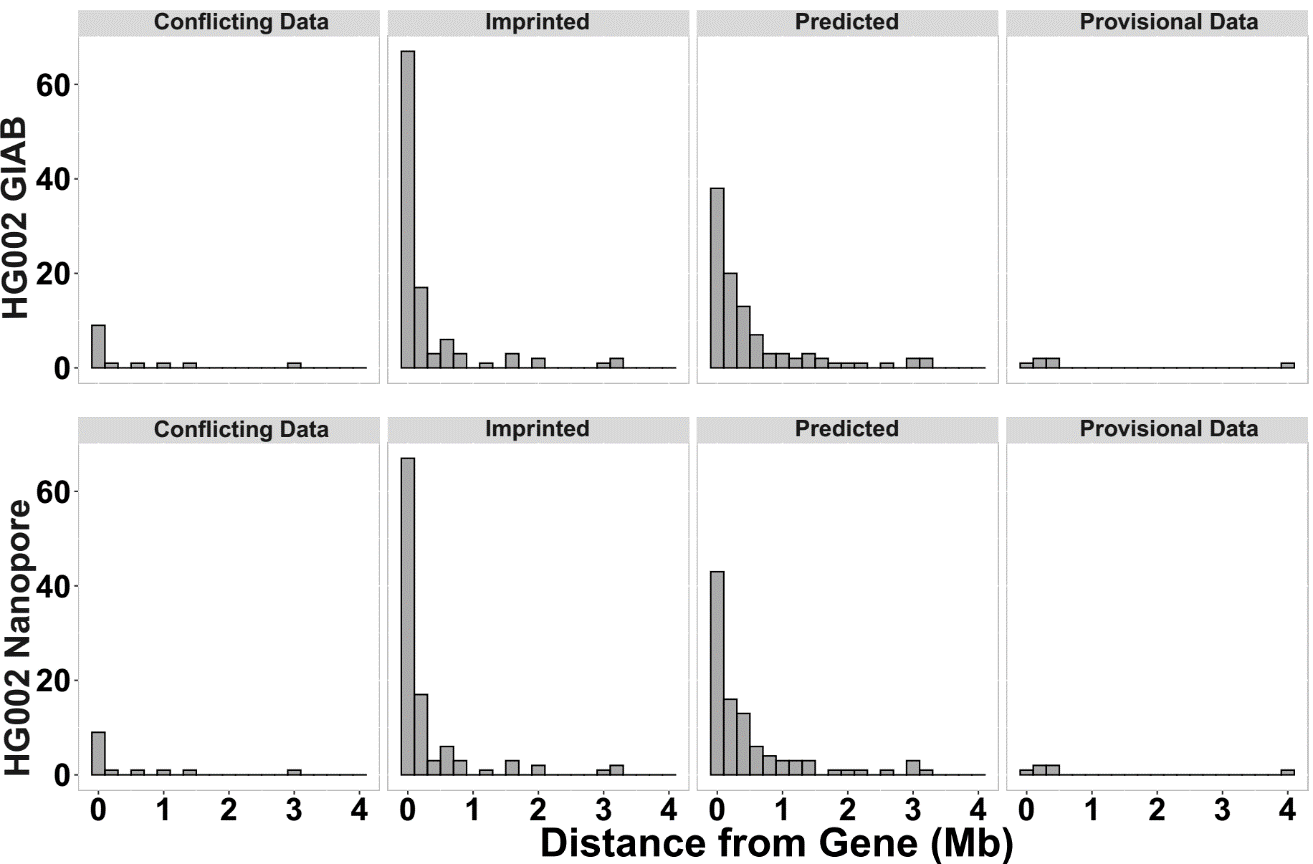

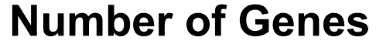


**Fig. S2:** Mapping DMRs to 4Mb window of the gene list from GeneImpring and the Cataloguoe of Imprinted Genes.

# References

1. Jain M, Koren S, Miga KH, Quick J, Rand AC, Sasani TA, et al. Nanopore sequencing and assembly of a human genome with ultra-long reads. Nat Biotechnol [Internet]. 2018;36:338–45. Available from: https://doi.org/10.1038/nbt.4060

2. Simpson JT, Workman RE, Zuzarte PC, David M, Dursi LJ, Timp W. Detecting DNA cytosine methylation using nanopore sequencing. Nat Methods [Internet]. Nature Publishing Group, a division of Macmillan Publishers Limited. All Rights Reserved.; 2017;14:407. Available from: https://doi.org/10.1038/nmeth.4184

3. Ni P, Huang N, Zhang Z, Wang D-P, Liang F, Miao Y, et al. DeepSignal: detecting DNA methylation state from Nanopore sequencing reads using deep-learning. Bioinformatics [Internet]. 2019;35:4586–95. Available from: https://doi.org/10.1093/bioinformatics/btz276

4. Oxford Nanopore Technologies. Megalodon. GitHub [Internet]. 2020. Available from: https://github.com/nanoporetech/megalodon

5. Li H. Minimap2: pairwise alignment for nucleotide sequences. Bioinformatics [Internet]. 2018;34:3094–100. Available from: https://doi.org/10.1093/bioinformatics/bty191

6. Li H, Handsaker B, Wysoker A, Fennell T, Ruan J, Homer N, et al. The Sequence Alignment/Map format and SAMtools. Bioinformatics [Internet]. 2009;25:2078–9. Available from: https://doi.org/10.1093/bioinformatics/btp352

7. Zook JM, Catoe D, McDaniel J, Vang L, Spies N, Sidow A, et al. Extensive sequencing of seven human genomes to characterize benchmark reference materials. Sci Data [Internet]. 2016;3:160025. Available from: https://doi.org/10.1038/sdata.2016.25

8. De Coster W, De Rijk P, De Roeck A, De Pooter T, D’Hert S, Strazisar M, et al. Structural variants identified by Oxford Nanopore PromethION sequencing of the human genome. Genome Res. Cold Spring Harbor Lab; 2019;29:1178–87.

9. Li H. Aligning sequence reads, clone sequences and assembly contigs with BWA-MEM. arXiv Prepr arXiv13033997. 2013;

10. Luo R, Wong C-L, Wong Y-S, Tang C-I, Liu C-M, Leung C-M, et al. Exploring the limit of using a deep neural network on pileup data for germline variant calling. Nat Mach Intell [Internet]. 2020;2:220–7. Available from: https://doi.org/10.1038/s42256-020-0167-4

11. Kim S, Scheffler K, Halpern AL, Bekritsky MA, Noh E, Källberg M, et al. Strelka2: fast and accurate calling of germline and somatic variants. Nat Methods [Internet]. 2018;15:591–4. Available from: https://doi.org/10.1038/s41592-018-0051-x

12. Auton A, Abecasis GR, Altshuler DM, Durbin RM, Abecasis GR, Bentley DR, et al. A global reference for human genetic variation. Nature [Internet]. 2015;526:68–74. Available from: https://doi.org/10.1038/nature15393

13. Zhao H, Sun Z, Wang J, Huang H, Kocher J-P, Wang L. CrossMap: a versatile tool for coordinate conversion between genome assemblies. Bioinformatics. Oxford University Press; 2014;30:1006–7.

14. Martin M, Patterson M, Garg S, O Fischer S, Pisanti N, Klau GW, et al. WhatsHap: fast and accurate read-based phasing. bioRxiv [Internet]. 2016;85050. Available from: https://www.biorxiv.org/content/10.1101/085050v2.full

15. Court F, Tayama C, Romanelli V, Martin-Trujillo A, Iglesias-Platas I, Okamura K, et al. Genome-wide parent-of-origin DNA methylation analysis reveals the intricacies of human imprinting and suggests a germline methylation-independent mechanism of establishment. Genome Res. Cold Spring Harbor Lab; 2014;24:554–69.

16. Joshi RS, Garg P, Zaitlen N, Lappalainen T, Watson CT, Azam N, et al. DNA Methylation Profiling of Uniparental Disomy Subjects Provides a Map of Parental Epigenetic Bias in the Human Genome. Am J Hum Genet [Internet]. Elsevier; 2016;99:555–66. Available from: https://doi.org/10.1016/j.ajhg.2016.06.032

17. Zink F, Magnusdottir DN, Magnusson OT, Walker NJ, Morris TJ, Sigurdsson A, et al. Insights into imprinting from parent-of-origin phased methylomes and transcriptomes. Nat Genet [Internet]. 2018;50:1542–52. Available from: https://doi.org/10.1038/s41588-018-0232-7
