# Supplementary material for: Megabase-scale methylation phasing using nanopore long reads and NanoMethPhase
Source: Genome Biol. 2021 Feb 22;22:68. doi: 10.1186/s13059-021-02283-5 (PMC7898412; doi:10.1186/s13059-021-02283-5)

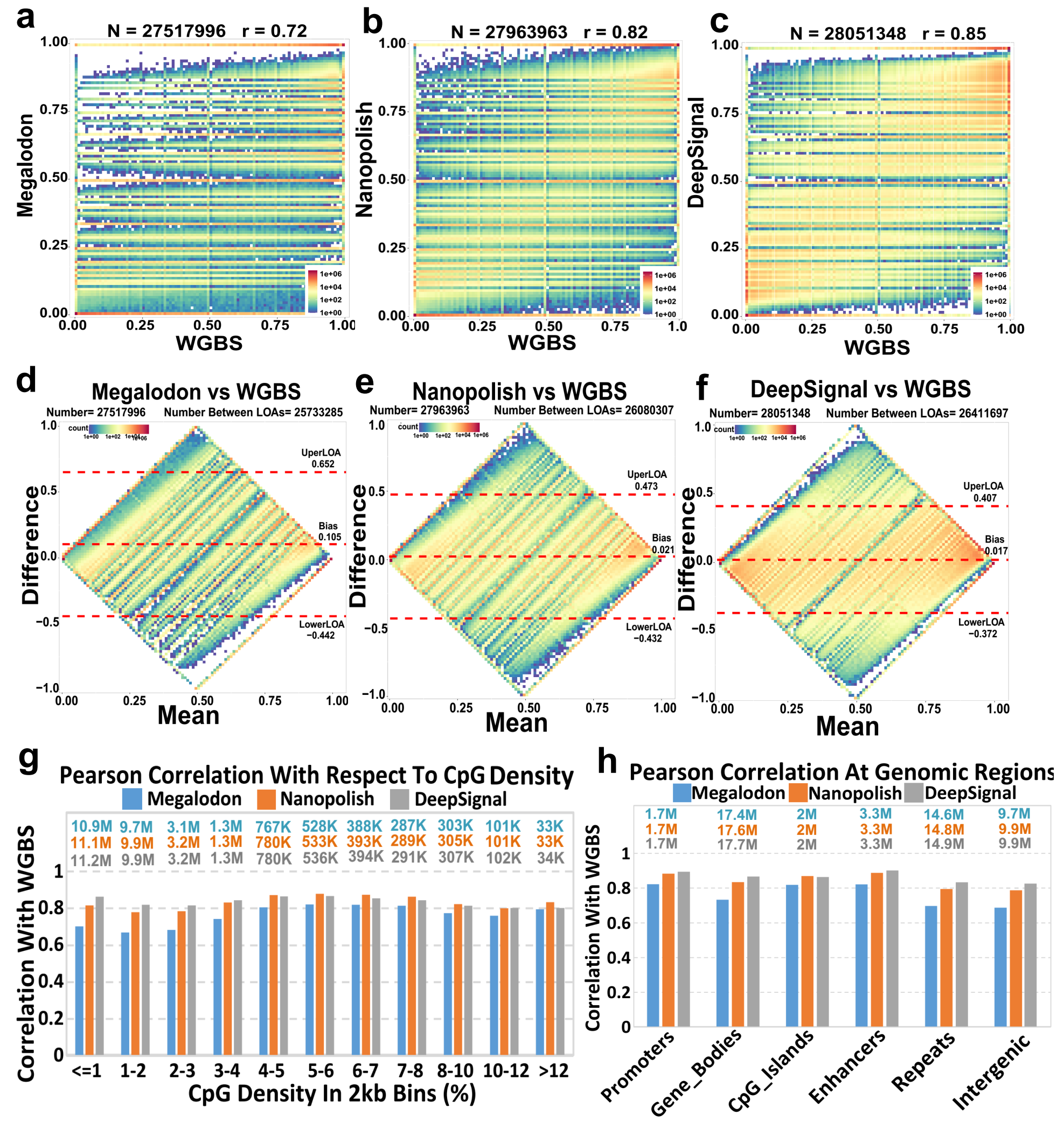


**Additional File 2: Supplementary Figures**

**Fig. S1.** Comparison of CpG methylation detected by nanopore with WGBS. (a,b,c) Correlation between CpG methylation frequencies detected using each tool from nanopore data with WGBS. This correlations represent at all detected CpGs common with WGBS using each tool. (d,e,f) Bland–Altman plot between CpG methylation frequencies detected using each tool from nanopore data with WGBS. DeepSignal represented highest agreement with WGBS with lowest bias, narrower limits of agreement (LOAs), and more points in between of the LOAs. (g) Correlations with WGBS in respect to CpG density. For this analysis, we binned human genome (hg38) into 2kb bins and calculated percent of CpG at each 2kb bin. (h) Correlations with WGBS at different genomic regions. For gene body regions we included all genes in GeneCode v34 (pseudogenes included). Promoters defines as 1000bp up-stream and 200bp downstream of transcription start site. The numbers on top of the bars represent number of CpGs each method had in common with WGBS.

**Fig. S2**. Mapping results of uncommon CpGs between nanopore and WGBS to genomic features (this uncommon sites also captures abundant CpGs detected by nanopore but not WGBS). (a) UpSet plot representing number of CpGs mapped to each feature and their intersections. For ease in visualization only intersections with >2000 CpGs are shown. (b) CpGs mapped to each satellite repeat type.


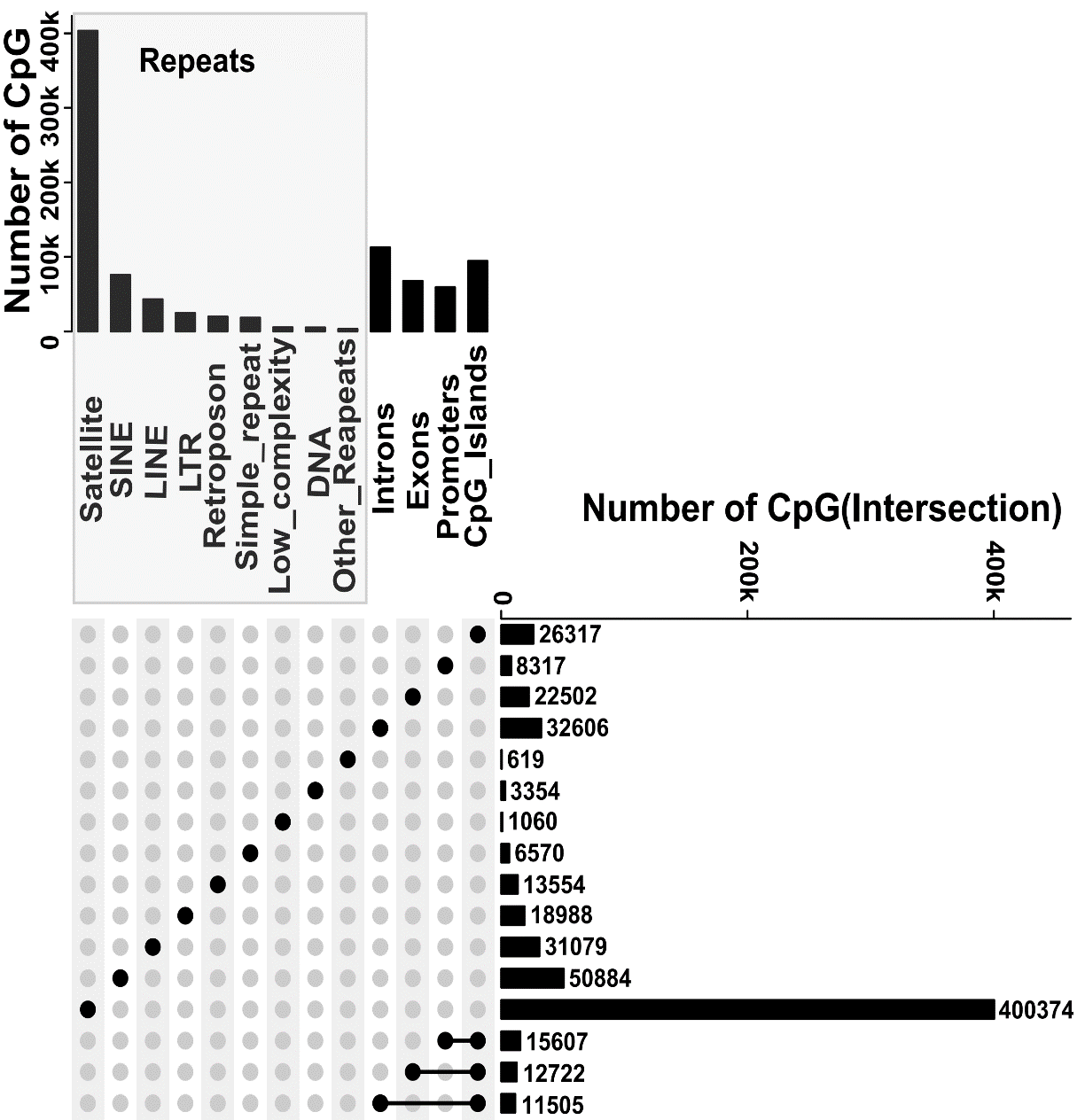

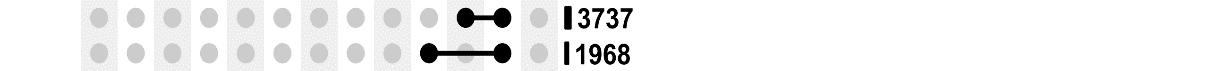

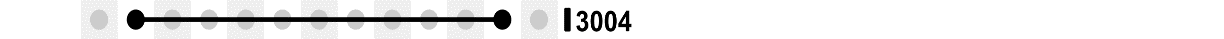

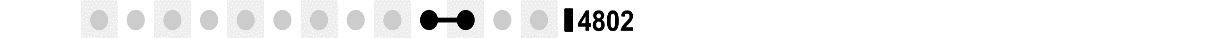

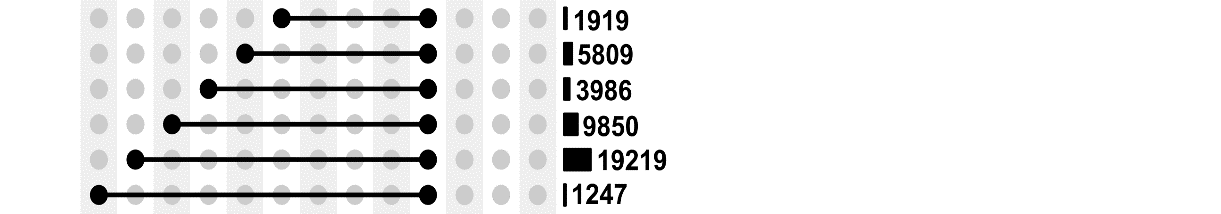

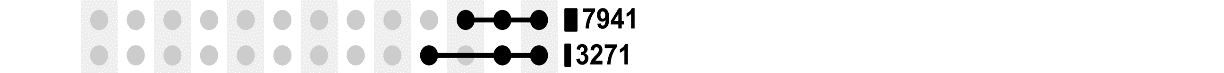

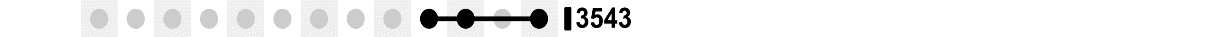

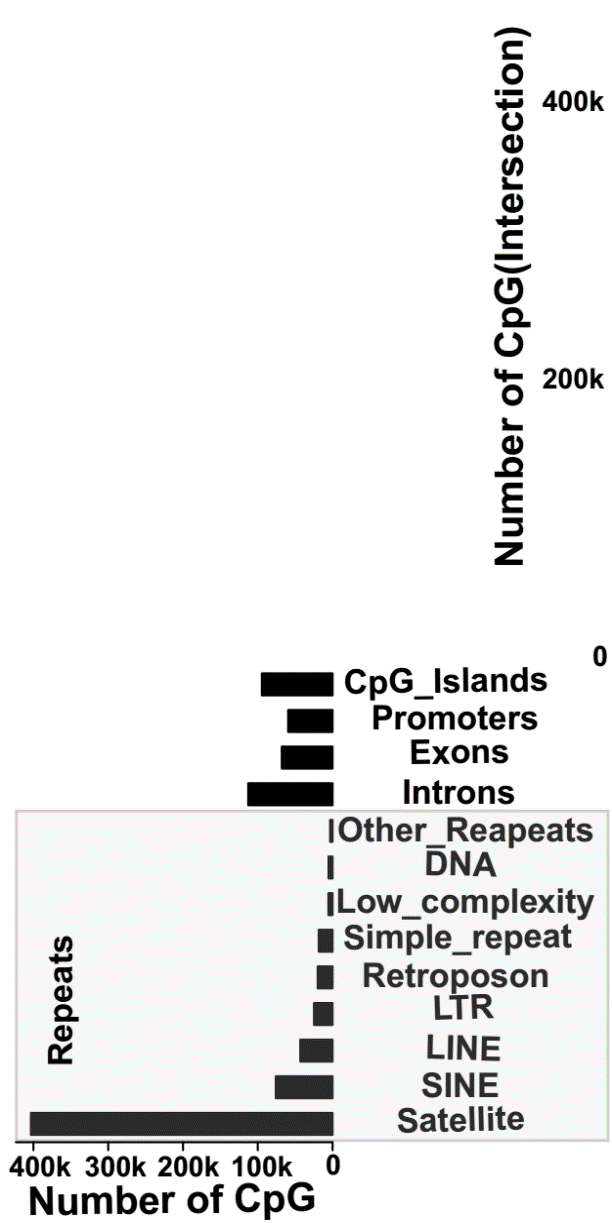

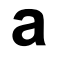

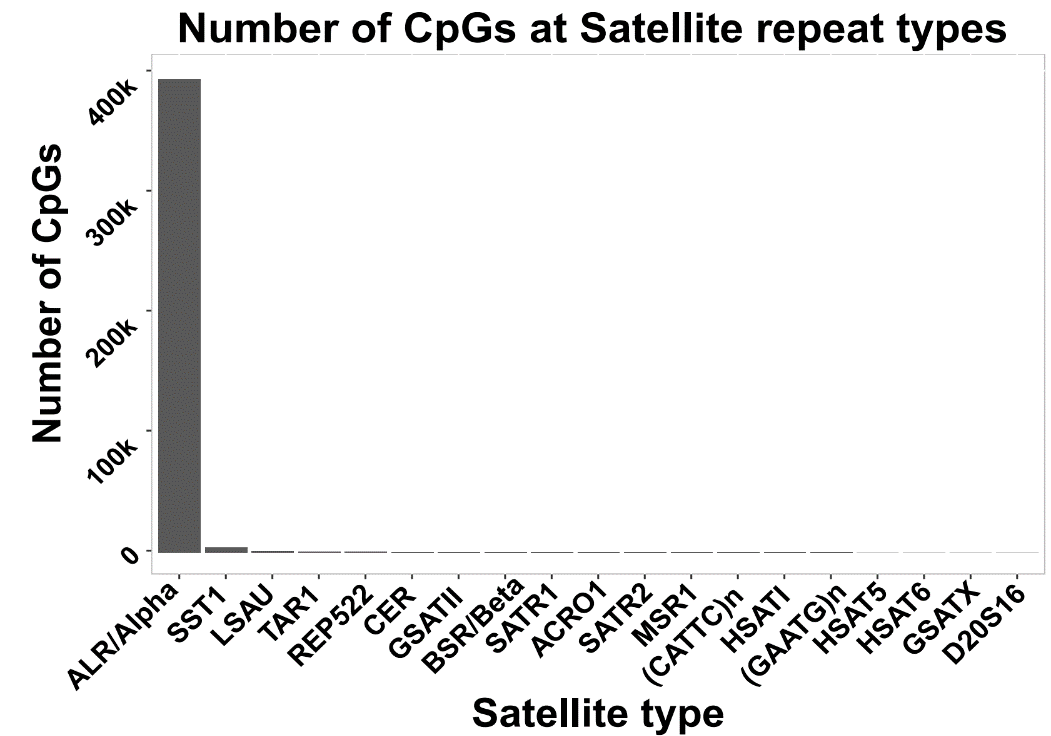

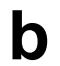


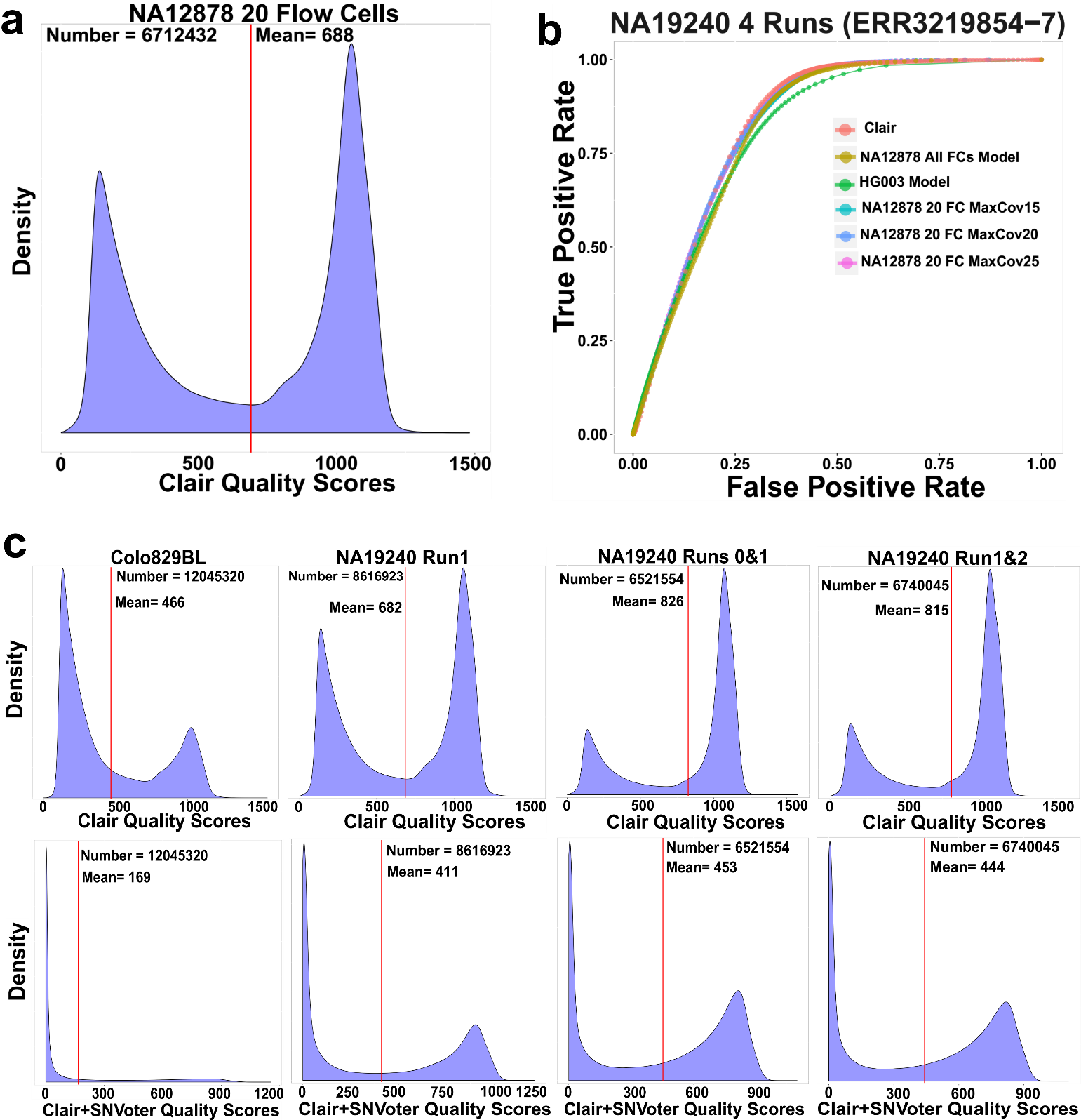


**Fig. S3.** Distribution of variant call qualities before and after normalization using SNVoter. (a) Clair variant call quality distribution for NA12878 20 flow cells sample. The distribution is used to obtain optimal threshold for quality filtering of Clair’s results in this sample (<https://github.com/HKU-BAL/Clair>). (b) ROC curve using various threshold for Clair qualities and normalized qualities (Clair+SNVoter) on NA19240 4 run sample (75x) showing no advantage when using SNVoter on high coverage data. (c) Variant call quality distributions before and after applying SNVoter on Clair’s results for 4 different datasets with coverages from 10x to 40x.


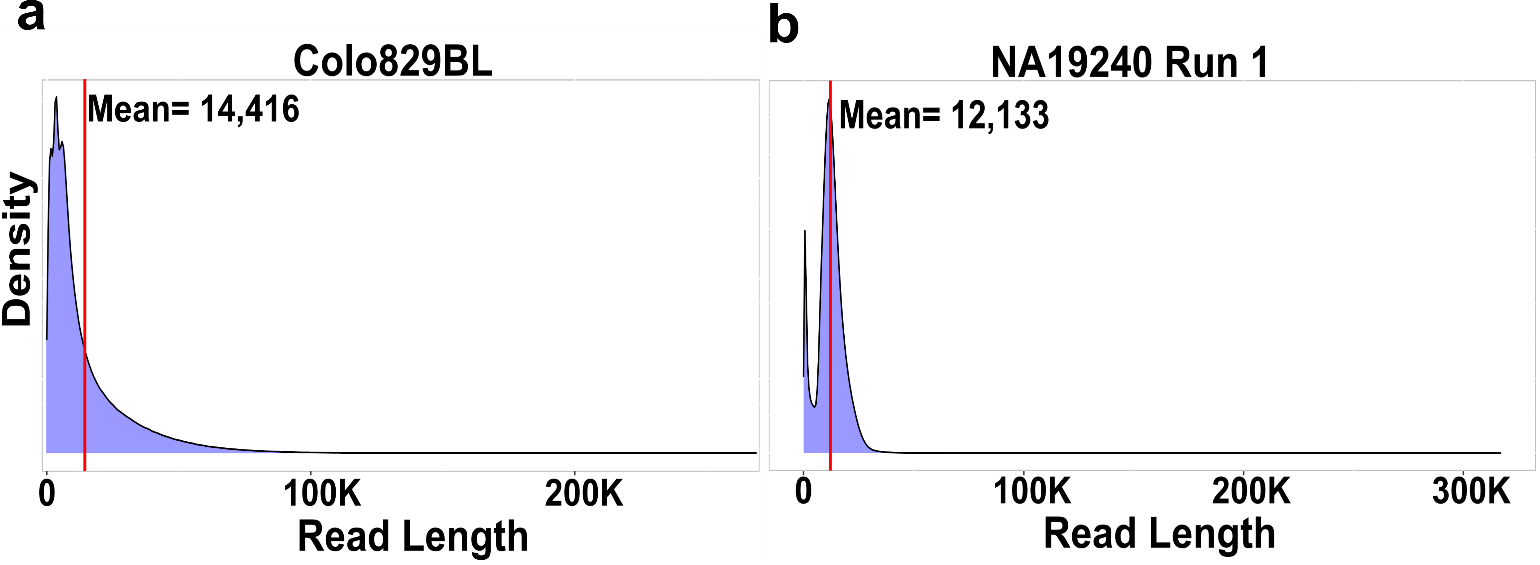


**Fig. S4**. Read length distribution for (a) Colo829BL and (b) NA19240 run 1 samples.


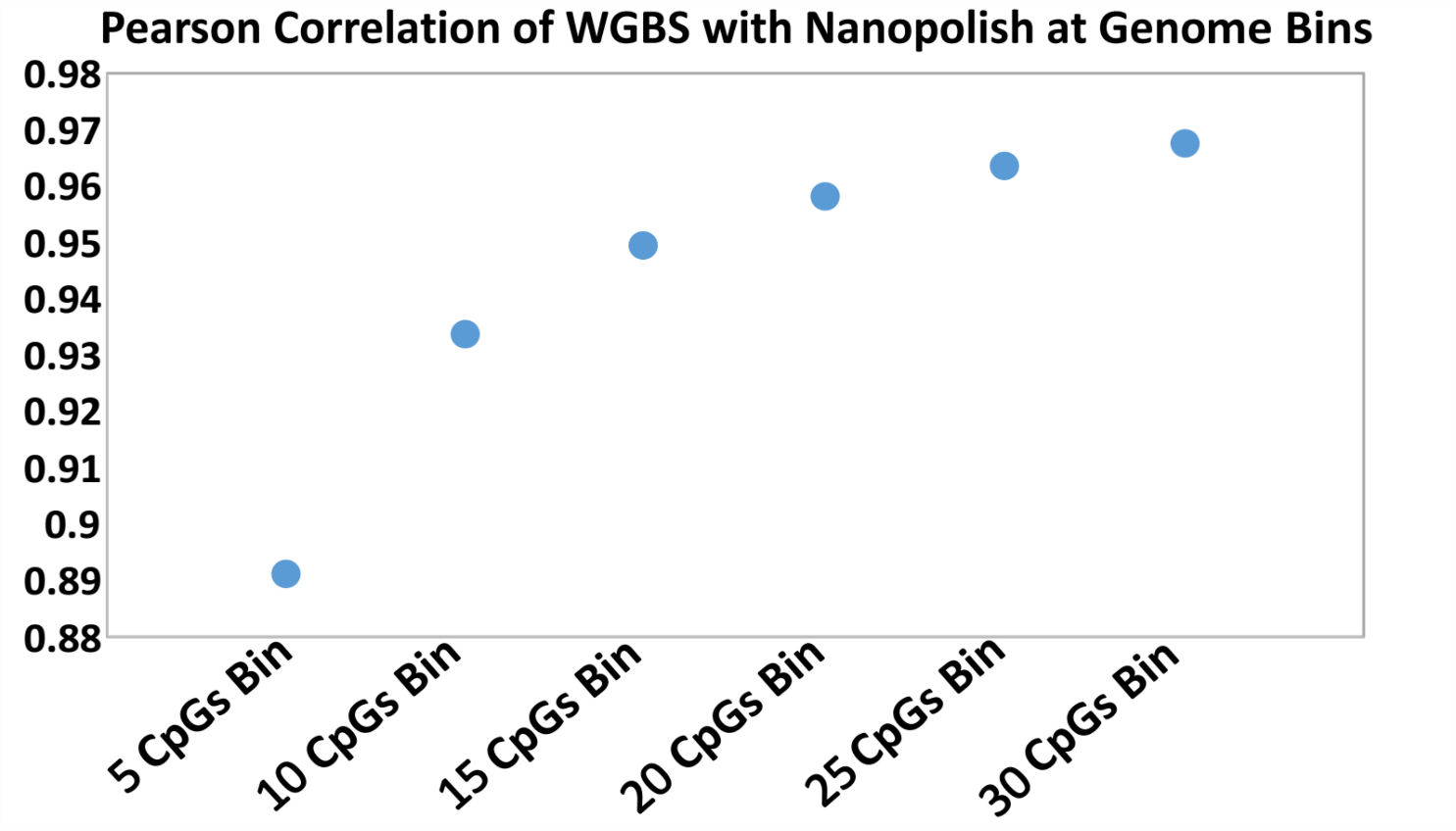


**Fig. S5**. Pearson correlation of average CpG methylation from WGBS and nanopore sequencing at genomic bins with constant number of CpGs.


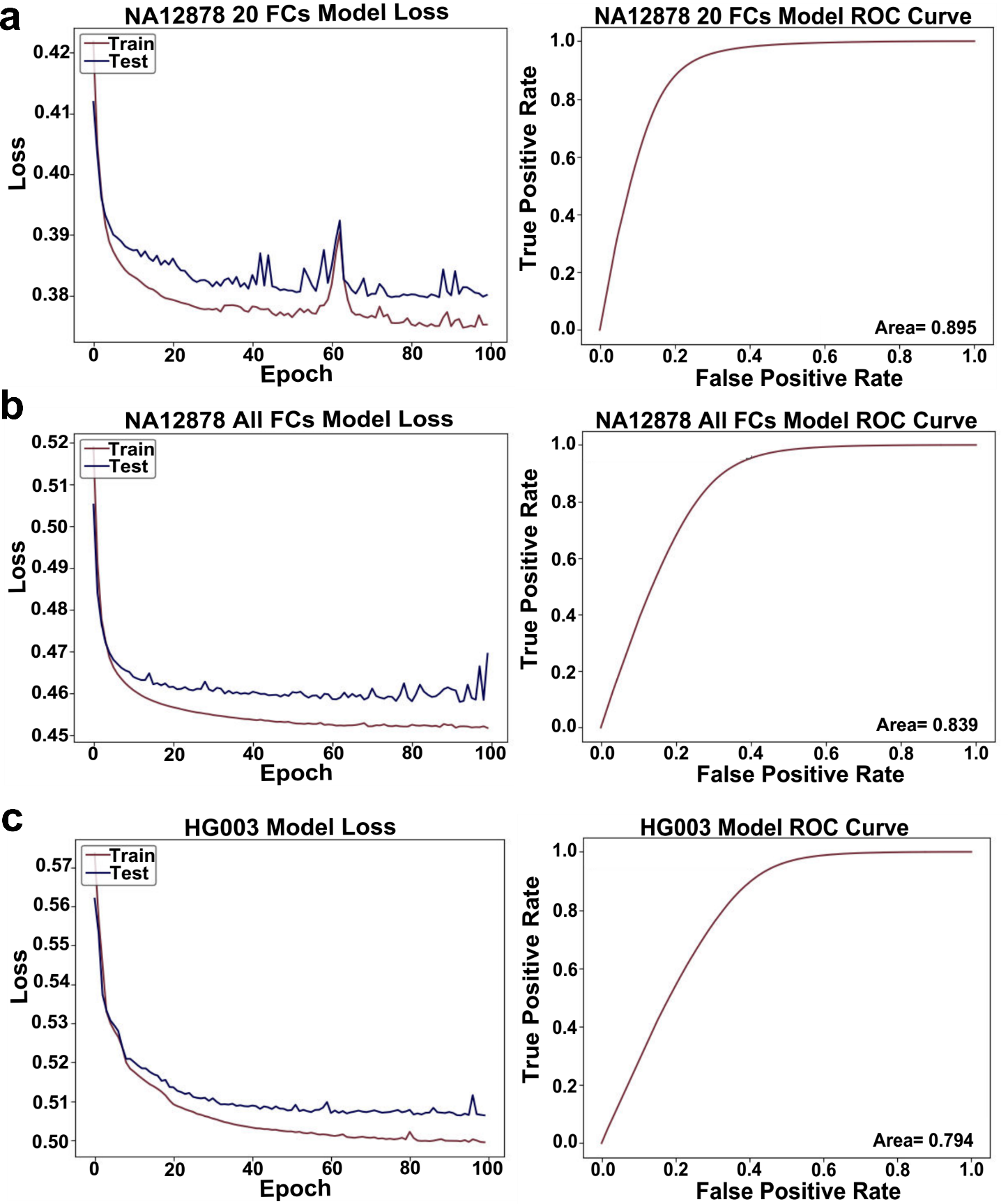


**Fig. S6**. Model training to improve SNV calling using Clair. Model training Loss and ROC curves for the three different models trained using 3 datasets to capture different coverages. (a) NA12878 20 flow cells (20 FCs, 24x coverage) model. (b) NA12878 all FCs (44x coverage). (c) HG003 (80x coverage).


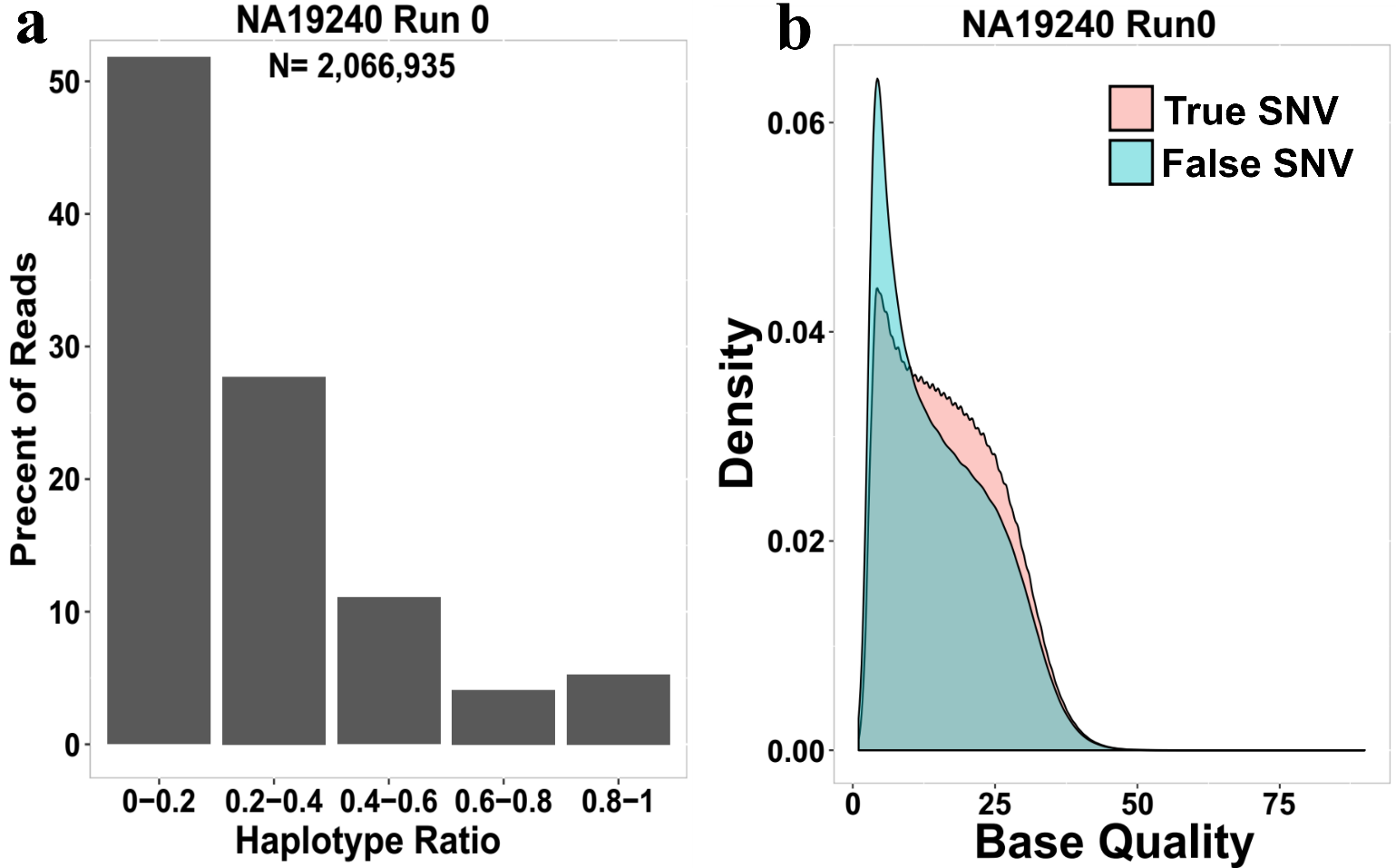


**Fig. S7**. (a) Histogram showing haplotype ratios for NA19240 run 0 sample (number of SNVs from HP1 over number of reads from HP2 for the same read and vice versa). (b) Distribution of true and false positive SNV calls for NA19240 run 0 sample which could pass quality filtering after applying SNVoter on Clair’s SNV calls.

**Fig. S8**. ROC curves for SNV calling in Colo829BL sample aligned with winnowmap and minimap2.


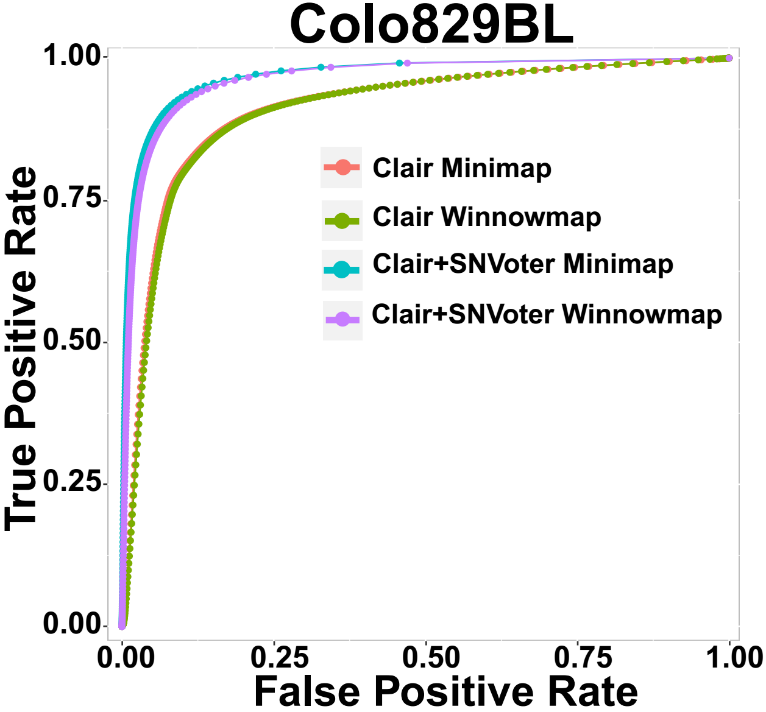

Supplement: Supplementary file 2 — Additional file 2. Contains additional figures for the paper, such as more correlation analysis for comparison of nanopore methylation call with WGBS and mapping of nanopore specific CpG methylation calls to genomic regions, supporting figures for SNV improvement using SNVoter, Read length distribution for Colo829BL and NA19240, Model training plots for SNVoter, etc. [file 13059_2021_2283_MOESM2_ESM.docx]
