# Supplementary material for: Megabase-scale methylation phasing using nanopore long reads and NanoMethPhase
Source: Genome Biol. 2021 Feb 22;22:68. doi: 10.1186/s13059-021-02283-5 (PMC7898412; doi:10.1186/s13059-021-02283-5)
Supplement: Supplementary file 12 — Additional file 12. Review history. [file 13059_2021_2283_MOESM12_ESM.docx]

Review History

**First round of review**

**Reviewer 1**

**Were you able to assess all statistics in the manuscript, including the appropriateness of statistical tests used?**

Yes, and I have assessed the statistics in my report.

**Were you able to directly test the methods?**

No

**Comments to author:**

Paper title: " Megabase scale methylation phasing using Nanopore long reads and NanoMethPhase". By Akbari et al.

Manuscript ID: GBIO-D-20-01400

comments:

1. The tool and analyses provided in the paper could be of high value in the field in future as Nanopore sequencing is gaining popularity. However, I feel several modification and addition will be required.

2. The introduction is generally well written. However, I would like more background of Nanopore sequencing and description of how it differs to the bisulfite sequencing which has been and still is the gold standard in the field (with appropriate referencing).

3. The authors described the comparison with WGBS and 27K array data and in my opinion this is perhaps the most important piece of data. However, I feel additional analysis need to be added. What will be important is to have Nanopore methylation data and WGBS methylation data from same samples and then compare them directly against each other (show correlation in XY plot for all regions investigated, a Bland Altman plot to show the differences to complement results.

4. The authors need to comment and describe how Nanopore methylation call compares to bisulfite methylation when it comes to regions which are CpG rich vs CpG poor. It's likely the accuracy of CpG rich region might be lower in Nanopore than bisulfite based assay (there are good evidence from previous studies).

5. It will be useful to also comment on the ability of detecting DMRs and the comparative performance.

6. Is there other regions in the genome (say enhancers or intergenic regions) that perform differently in their methylation calls in their analysis ?

7. I tried to find detailed documentation of the tool and description of analyses step for this paper. I think it's not enough and this should be improved. The idea should be to allow bench scientists to easily utilise this tool to analyse Nanopore methylation sequencing data. The authors need to provide a separate file with step-by-step guideline for bench scientists to perform the analyses described. An example dataset to perform these analyses should also be provided (could just be one chromosome for example, the point is to provide real example data to try).

8. The discussion is mainly rephrasing of results instead of a critical assessment of the current literature and placing their findings in regards to that. The authors should revisit this section to make it more meaningful.

**Reviewer 2**

**Were you able to assess all statistics in the manuscript, including the appropriateness of statistical tests used?**

Yes, and I have assessed the statistics in my report.

**Were you able to directly test the methods?**

Yes

**Comments to author:**

Dear authors,

The manuscript by Akbari et al. is interesting and performs a good series of investigations using Nanopore sequencing data. They used nanopolish to detect CpG methylation along with Clair for SNV calling. They also trained a neural network to further refine SNV calls made by Clair, and demonstrate their toolkit SNVoter and NanoMethPhase on NA19240, GM12878 and another B-cell line.

I liked their approach in general, wherein they combine the multiple tools that are independently available into a streamlined workflow. I also appreciated that the authors included their run commands in the supplementary files (very good practice). I have a few minor comments:

1. The basecalling versions are somewhat inconsistent among different data. While this is not a major issue, I would highly recommend that the authors re-basecall the data using Guppy 4.0.11 or Guppy 4.2.2. The accuracy improvements should yield better SNV calling.

2. It could be also interesting to perform the same analysis but using the de novo methylation in Guppy that is now available.

3. Have the authors tried Winnowmap for mapping instead of minimap2? In the recent work from the Telomere-to-Telomere consortium, my impression was that the longer reads are better mapped by Winnowmap, especially in repeat-rich regions.

4. I may have missed this in the manuscript. What was the rationale for variable coverages being used during different steps, for example 12X during methylation, 24X during SNV calling, before using high coverage datasets during training?

5. Also, what was the rationaly for using HG003? I assume high-coverage?

6. It may also be interesting to perform a benchmarking test for NanoMethPhase and SNVoter using the Ashkenazi Trio since those are all long read nanopore data and (if I understood their paper correctly) from the same batch of sequencing. What do the authors think?

I send my best wishes to the authors.

**Authors Response**

**Point-by-point responses to the reviewers’ comments:**

Response to Reviewer Comments:

*Thank you for considering our manuscript entitled "Megabase scale methylation phasing using Nanopore long reads and NanoMethPhase" for resubmission to Genome Biology.*

*Based on the comments from reviewers, we performed more comparisons between nanopore methylation calls and whole genome bisulfite sequencing. We have also re-basecalled Colo829BL and NA19240 samples with the guppy v4.2.2 and compare the results with previous data basecalled using older version of guppy. We detected allele-specific methylation using the Ashkenazi trio which all three samples sequenced using nanopore sequencing through The Genome in a Bottle Consortium.*

Reviewer #1

1. The tool and analyses provided in the paper could be of high value in the field in future as Nanopore sequencing is gaining popularity. However, I feel several modification and addition will be required.

*We thank the reviewer for their positive evaluation of the presented work. We incorporated the modification listed below.*

2. The introduction is generally well written. However, I would like more background of Nanopore sequencing and description of how it differs to the bisulfite sequencing which has been and still is the gold standard in the field (with appropriate referencing).

*We have revisited the introduction section to cover the explanations regarding both WGBS and Nanopore sequencing and how they work and differ.*

*“The current gold standard approach to study DNA methylation is whole-genome bisulfite sequencing (WGBS) [7,8]. Discrimination of methylated and unmethylated CpGs in WGBS is based on bisulfite conversion. During bisulfite treatment unmethylated cytosine converted to uracil which then replaced by thymidine through downstream PCR reactions prior to short-read sequencing. Subsequent short-read sequencing and mapping of reads to the reference genome can detect DNA methylation genome-wide [7,8].*

*In nanopore sequencing, as nucleic acids are propelled through a protein nanopore embedded in an electrically-resistant membrane, the chemical composition of the approximately five nucleotides (5-mers) present in the narrowest region of the pore define the measurable current signal across the membrane (pore version R9.4) [13]. These distinctive signal characteristics are interpretable by a trained artificial neural network during typical basecalling. A similar approach is used to call 5-methylcytosine at CpG sites by tools such as Megalodon [14], Nanopolish [10], DeepSignal [15], DeepMod [16], and SignalAlign [17]. Therefore, in nanopore sequencing both DNA sequences and modifications are detectable using raw signal information and there is no need for bisulfate conversion and PCR amplification prior to sequencing [16,18].”*

(Comments 3, 4, 6): 3. The authors described the comparison with WGBS and 27K array data and in my opinion this is perhaps the most important piece of data. However, I feel additional analysis need to be added. What will be important is to have Nanopore methylation data and WGBS methylation data from same samples and then compare them directly against each other (show correlation in XY plot for all regions investigated, a Bland Altman plot to show the differences to complement results. And 4. The authors need to comment and describe how Nanopore methylation call compares to bisulfite methylation when it comes to regions which are CpG rich vs CpG poor. It's likely the accuracy of CpG rich region might be lower in Nanopore than bisulfite based assay (there are good evidence from previous studies). And 6. Is there other regions in the genome (say enhancers or intergenic regions) that perform differently in their methylation calls in their analysis?

*The section “Benchmarking of Nanopore Methylation Calling Software” was revisited to include the correlation analysis of methylated sites from WGBS and Nanopore called using the three softwares.*

*“The correlation with WGBS was also evaluated based on CpG density and also at each genomic regions (additional file 2, figure 1g,h). These analyses showed that the correlation is lower at low and high density CpG sites (<4% CpG per 2kb and >8% CpG per 2kb) and deepsignal works better at low dense CpG sites while nanopolish gives better correlation at high density CpG regions. Moreover, the best correlation with WGBS obtained at enhancers and promoters, while repeats and intergenic regions had the lowest correlation .In agreement with correlation analysis based on CpG density, nanopolish showed slightly better correlation at CpG islands while DeepSignal worked better in other regions.”*

5. It will be useful to also comment on the ability of detecting DMRs and the comparative performance.

*The highest correlation between Nanopore and WGBS obtained at CpG ratio (number of CpGs/ICR length) of 4-8%. We observed that Known ICRs with CpG ratio 4-8 tend to be more detected. However, this is mainly observed in lower coverage samples (Colo829BL and NA19240). Overall, 28 of the known ICRs have CpG ratio 4-8% and 15 less than 4% and more than 8%. Detected Known ICRs in each sample are as follow:*

7. I tried to find detailed documentation of the tool and description of analyses step for this paper. I think it's not enough and this should be improved. The idea should be to allow bench scientists to easily utilise this tool to analyse Nanopore methylation sequencing data. The authors need to provide a separate file with step-by-step guideline for bench scientists to perform the analyses described. An example dataset to perform these analyses should also be provided (could just be one chromosome for example, the point is to provide real example data to try).

*The source repositories provided with the initial submission of the manuscript contained the “quick start” guides that was meant to be a command line usage resource (https://github.com/vahidAK/NanoMethPhase and https://github.com/vahidAK/SNVoter). The commands that were used to process the data of the manuscript was supplied in supplementary file 1. We updated the README file in order to display the help output, documentation and a full tutorial with the steps to reach phased reads from scratch. A short example set is also included, comprising of NA19240 1Mb of chromosome 21 and can be found in the “Example_Data” folder of the repository.*

8. The discussion is mainly rephrasing of results instead of a critical assessment of the current literature and placing their findings in regards to that. The authors should revisit this section to make it more meaningful.

*The discussion section was revisited to include more comparison to current literature.*

Reviewer #2

The manuscript by Akbari et al. is interesting and performs a good series of investigations using Nanopore sequencing data. They used nanopolish to detect CpG methylation along with Clair for SNV calling. They also trained a neural network to further refine SNV calls made by Clair, and demonstrate their toolkit SNVoter and NanoMethPhase on NA19240, GM12878 and another B-cell line.

I liked their approach in general, wherein they combine the multiple tools that are independently available into a streamlined workflow. I also appreciated that the authors included their run commands in the supplementary files (very good practice). I have a few minor comments:

1. The basecalling versions are somewhat inconsistent among different data. While this is not a major issue, I would highly recommend that the authors re-basecall the data using Guppy 4.0.11 or Guppy 4.2.2. The accuracy improvements should yield better SNV calling.

*We re-basecalled Colo829BL and NA19240 Run 1 sample using Guppy 4.2.2 as these two samples went through the whole pipeline (SNV detection using Clair, SNV impprovment using SNVoter, and methylation phasing using NanoMethPhase) to first investigate the advantage of using new version of Guppy. Because deposited data are usually basecalled using older versions and basecalling itself is a computationally expencive and timely process, we included this analyses as supplementary data in additional file 1 to readers have sense of ASM detection using older and newer version.*

*“Oxford Nanopore Technologies constantly releases new base caller versions. To investigate if using newer version of base caller improves ASM detection we re-basecalled Colo829BL and NA19240 samples using Guppy v4.2.2.*

*Variant calling using Clair and then improving Clair’s calls using SNVoter resulted in detection more true positive SNVs but also more false positive and only improved recall (Table 2). Using the new version*

*Table 2: Different metrics for SNV calls using Clair and Clair + SNVoter and newest guppy version.*

*After methylation calling and phasing, DMA between haplotypes was performed. 876 (~77% in common with base calling using old guppy) DMRs detected in Colo829BL, 2090 (~89% in common with base calling using old guppy) in NA19240 pure nanopore, and 1965 (~82% in common with base calling using old guppy) in NA19240 trio phasing. We mapped detected DMRs to known and novel DMRs reported by three previous studies (Table 3). This resulted in detection of very few more reported ICRs.*

*Table 3: Mapping DMRs to reported known and novel ICRs.*

2. It could be also interesting to perform the same analysis but using the de novo methylation in Guppy that is now available.

*Basically, Megalodon is a reference anchored guppy methylation caller and uses the same model as Guppy. Moreover, Guppy does not output methylation calls such that to be used in our pipeline. Older versions of guppy store methylation calls in fast5 format and in newest version stores methylation information in a bam format file.*

3. Have the authors tried Winnowmap for mapping instead of minimap2? In the recent work from the Telomere-to-Telomere consortium, my impression was that the longer reads are better mapped by Winnowmap, especially in repeat-rich regions.

*The benchmarking of mapping tools are out of scope. The tools provided are compatible with any mapping tools. So it's up to the user to make sure that the mapping is appropriate. Our sequencing pipeline already QC testing that is compliant with minimap2. However, we realigned Colo829BL sample using Winnowmap and compared the SNV detection when aligned with minimap. The results, as shown below, were fairly similar and no improvement observed. Winnowmap improves alignment mostly in ultra-long read while our sample’s read length is much lower than telomere-to-telomere consortium data, so this might be the reason no improvement was observed.*

(Comments 4 and 5): 4. I may have missed this in the manuscript. What was the rationale for variable coverages being used during different steps, for example 12X during methylation, 24X during SNV calling, before using high coverage datasets during training? And 5. Also, what was the rationaly for using HG003? I assume high-coverage?

*We wanted to investigate the performance of methylation calling at lower coverage because we also wanted to investigate ASM detection in low coverage data. On the other hand, for development of our tool, SNVoter, we used 24 coverage to have enough features for the model. We empirically observed this coverage can recapitulate features at different regions. Subsequently, as we did not observed improvement in SNV detection using 24x model in high coverage data, we speculate a model trained using higher coverage data might improve the SNV calling. However such models could not offer an improvement. The rational for HG003 was to train a model using a very high coverage data to see if it can improve SNV detection in high coverage data.*

6. It may also be interesting to perform a benchmarking test for NanoMethPhase and SNVoter using the Ashkenazi Trio since those are all long read nanopore data and (if I understood their paper correctly) from the same batch of sequencing. What do the authors think?

*NanoMethPhase doesn't require the parental reads and in a trio study where we aim to phase methylome in child we use parental SNVs for phasing. Therefore, it won’t be an issue if the parental and child’s are note from the same sequencing batch. To investigate the performance of phasing when SNV data for all samples in trio come from nanopore sequencing we used Ashkenazi trio. Moreover, we compared this phasing for Ashkenazi trio with phasing of this trio using high confidence SNV calls from the Genome in a Bottle which obtained using short-read sequencing. We included the results of this analyses in additional file 1 section 5. This analyses demonstrated detection of most of the known ICRs (86%) and high commonalities in terms of DMRs and phased reads to maternal and paternal haplotypes between phasing using SNVs detected from nanopore data for trio sample and phasing using SNVs from GIAB which detected using sort-read data.*

*“Ashkenazi trio base called reads for all three samples along with promethION raw fast5 files for HG002 were downloaded from GIAB GitHub and GIAB BioProject in SRA (https://github.com/genome-in-a-bottle/giab_data_indexes and PRJNA200694). Ahkenazi trio has base called nanopore sequencing data publically available. Moreover, there are publically available variant call data detected using Illumina short-read sequencing for this trio.*

*We phased reads in son (HG002) in two ways. First, where all SNVs in trio detected from nanopore data via Clair (SNVoter was not used as the data have high coverage). Second, where SNVs for the trio are coming from publically available variant call data detected using sort-read sequencing.*

*In phasing using SNVs detected from nanopore data for the trio, 1.62M reads were assigned to the maternal and 1.62M reads to the paternal haplotype and in phasing using SNVs from GIAB database 1.44M reads were assigned to the maternal and 1.44M reads to the paternal haplotype (1.38M reads were in common in maternal and 1.38M in paternal).*

*We detected 1,812 DMRs when reads were phased using GIAB variant call files and 2,066 when phased using variant detected from nanopore in trio (1,726 were overlapped). DMRs were mapped to known and novel ICRs reported by the previous studies (Table 4). They also mapped to 4Mb window around known imprinted genes (GeneImprint http://www.geneimprint.com/ and the catalogue of imprinted genes http://igc.otago.ac.nz/) (Figure 2).*

*Table 4 : Mapping DMRs to reported known and novel ICRs.*

**Second round of review**

**Reviewer 1**

The authors did address the comments raised in the revised manuscript.

**Authors Response**

**Point-by-point responses to the reviewers’ comments:**

Reviewers comments:

1- Reviewer #1: The authors did address the comments raised in the revised manuscript.

*We thank the reviewer for the constructive comments on our manuscript.*

2- The authors have addressed the comment of “ The basecalling versions are somewhat inconsistent among different data. While this is not a…… “ well with new basecalling data (table 2 and 3). However, I feel in the main manuscript these results were described with enough detail and clarity. Could the authors revisit this section and describe these new results with more details.

*We have discussed the results in the manuscript under the "Autosomal DMRs Mapped to Known ICRs and Known Imprinted Genes" section (yellow highlight).*

*"As ONT frequently releases new versions of Guppy with claimed higher accuracy, we aimed to investigate the effect of a new version of the basecaller on ASM detection (Additional file 1: Section 4). We re-basecalled Colo829BL and NA19240 run 1 using guppy v4.2.2. Even though, more true positive SNVs were detected at the optimal threshold more false positives were also presented (Table 1. Additional file 1: table S2). Therefore, no overall improvement in the SNV detection was observed. However, using guppy v4.2.2 the detection of ICRs slightly improved and we could detect one more reported ICR [32,33,41] in NA19240 and 6 more in Colo829BL (Table 3. Additional file 1: table S3) and, on average, about 82% of detected DMRs in Guppy 4.2.2 were overlapped with DMRs detected with older Guppy."*

I agree with the authors on response 3 (reviewer 2), however, I think this point should be added in the discussion section about Winnowmap.

*We have discussed the results from Winnowmap in the discussion section (yellow highlight).*

*"Mnimap2 [47] is a widely used aligner for nanopore long read data, therefore we also investigated the possibility of improvement in SNV detection using the recently developed aligner for nanopore long reads, Winnowmap [48]. We compared SNV detection for Colo829BL aligned to hg38 using Winnowmap to Minimap2 and no improvement observed (Additional file 2: Fig. S8)."*
